# A machine-learning based bio-psycho-social model for the prediction of non-obstructive and obstructive coronary artery disease

**DOI:** 10.1007/s00392-023-02193-5

**Published:** 2023-04-01

**Authors:** Valeria Raparelli, Giulio Francesco Romiti, Giulia Di Teodoro, Ruggiero Seccia, Gaetano Tanzilli, Nicola Viceconte, Ramona Marrapodi, Davide Flego, Bernadette Corica, Roberto Cangemi, Louise Pilote, Stefania Basili, Marco Proietti, Laura Palagi, Lucia Stefanini, EVA Investigators, Claudio Tiberti, Federica Panimolle, Andrea Isidori, Elisa Giannetta, Mary Anna Venneri, Laura Napoleone, Marta Novo, Silvia Quattrino, Simona Ceccarelli, Eleni Anastasiadou, Francesca Megiorni, Cinzia Marchese, Enrico Mangieri, Gaetano Tanzilli, Nicola Viceconte, Francesco Barillà, Carlo Gaudio, Vincenzo Paravati, Guglielmo Tellan, Evaristo Ettorre, Adriana Servello, Fabio Miraldi, Andrea Moretti, Alessandra Tanzilli, Piergiovanni Mazzonna, Suleyman Al Kindy, Riccardo Iorio, Martina Di Iorio, Gennaro Petriello, Laura Gioffrè, Eleonora Indolfi, Gaetano Pero, Nino Cocco, Loredana Iannetta, Sara Giannuzzi, Emilio Centaro, Sonia Cristina Sergi, Pasquale Pignatelli, Daria Amoroso, Simona Bartimoccia, Salvatore Minisola, Sergio Morelli, Antonio Fraioli, Silvia Nocchi, Mario Fontana, Filippo Toriello, Eleonora Ruscio, Tommaso Todisco, Nicolò Sperduti, Giuseppe Santangelo, Giacomo Visioli, Marco Vano, Marco Borgi, Ludovica Maria Antonini, Silvia Robuffo, Claudia Tucci, Agostino Rossoni, Valeria Spugnardi, Annarita Vernile, Mariateresa Santoliquido, Verdiana Santori, Giulia Tosti, Fabrizio Recchia, Francesco Morricone, Roberto Scacciavillani, Alice Lipari, Andrea Zito, Floriana Testa, Giulia Ricci, Ilaria Vellucci, Marianna Vincenti, Silvia Pietropaolo, Camilla Scala, Nicolò Rubini, Marta Tomassi, Gloria Rozzi, Floriana Santomenna, Claudio Cantelmi, Giacomo Costanzo, Lucas Rumbolà, Salvatore Giarrizzo, Carlotta Sapia, Biagio Scotti, Giovanni Talerico, Danilo Toni, Anne Falcou, Louise Pilote, Amanpreet Kaur, Hassan Behlouli, Anna Rita Vestri, Patrizia Ferroni, Clara Crescioli, Cristina Antinozzi, Francesca Serena Pignataro, Tiziana Bellini, Giovanni Zuliani, Angelina Passaro, Brombo Gloria, Andrea Cutini, Eleonora Capatti, Edoardo Dalla Nora, Francesca Di Vece, Andrea D’Amuri, Tommaso Romagnoli, Michele Polastri, Alessandra Violi, Valeria Fortunato, Alessandro Bella, Salvatore Greco, Riccardo Spaggiari, Gerarda Scaglione, Alessandra Di Vincenzo, Roberto Manfredini, Alfredo De Giorgi, Roberto Carnevale, Cristina Nocella, Carlo Catalano, Iacopo Carbone, Nicola Galea, Marianna Suppa, Antonello Rosa, Gioacchino Galardo, Maria Alessandroni, Alessandro Coppola, Mariangela Palladino, Giulio Illuminati, Fabrizio Consorti, Paola Mariani, Fabrizio Neri, Paolo Salis, Antonio Segatori, Laurent Tellini, Gianluca Costabile

**Affiliations:** 1 Department of Experimental Medicine, Sapienza University of Rome, Rome, Italy; 2 Department of Translational Medicine, University of Ferrara, Via Luigi Borsari, 46, 44121 Ferrara, Italy; 3 Faculty of Nursing, University of Alberta, Edmonton, Canada; 4 University Center for Studies on Gender Medicine, University of Ferrara, Ferrara, Italy; 5 Department of Translational and Precision Medicine, Sapienza University of Rome, Rome, Italy; 6 Liverpool Centre for Cardiovascular Science, University of Liverpool and Liverpool Heart and Chest Hospital, Liverpool, UK; 7 Department of Computer Control and Management Engineering Antonio Ruberti, Sapienza University of Rome, Rome, Italy; 8 Department of Clinical, Internal, Anesthesiology and Cardiovascular Sciences, Umberto I Hospital, Sapienza University of Rome, Rome, Italy; 9 Centre for Outcomes Research and Evaluation, McGill University Health Centre Research Institute, Montreal, QC Canada; 10 Divisions of Clinical Epidemiology and General Internal Medicine, McGill University Health Centre Research Institute, Montreal, QC Canada; 11 Division of Subacute Care, IRCCS Istituti Clinici Scientifici Maugeri, Milan, Italy; 12 Department of Clinical Sciences and Community Health, University of Milan, Milan, Italy

**Keywords:** Ischemic heart disease, Non-obstructive coronary artery disease, Frailty, Gender, Cytokines, Inflammation, Machine learning

## Abstract

**Background:**

Mechanisms of myocardial ischemia in obstructive and non-obstructive coronary artery disease (CAD), and the interplay between clinical, functional, biological and psycho-social features, are still far to be fully elucidated.

**Objectives:**

To develop a machine-learning (ML) model for the supervised prediction of obstructive versus non-obstructive CAD.

**Methods:**

From the EVA study, we analysed adults hospitalized for IHD undergoing conventional coronary angiography (CCA). Non-obstructive CAD was defined by a stenosis < 50% in one or more vessels. Baseline clinical and psycho-socio-cultural characteristics were used for computing a Rockwood and Mitnitski frailty index, and a gender score according to GENESIS-PRAXY methodology. Serum concentration of inflammatory cytokines was measured with a multiplex flow cytometry assay. Through an XGBoost classifier combined with an explainable artificial intelligence tool (SHAP), we identified the most influential features in discriminating obstructive versus non-obstructive CAD.

**Results:**

Among the overall EVA cohort (*n* = 509), 311 individuals (mean age 67 ± 11 years, 38% females; 67% obstructive CAD) with complete data were analysed. The ML-based model (83% accuracy and 87% precision) showed that while obstructive CAD was associated with higher frailty index, older age and a cytokine signature characterized by IL-1β, IL-12p70 and IL-33, non-obstructive CAD was associated with a higher gender score (i.e., social characteristics traditionally ascribed to women) and with a cytokine signature characterized by IL-18, IL-8, IL-23.

**Conclusions:**

Integrating clinical, biological, and psycho-social features, we have optimized a sex- and gender-unbiased model that discriminates obstructive and non-obstructive CAD. Further mechanistic studies will shed light on the biological plausibility of these associations.

**Clinical trial registration:**

NCT02737982.

**Graphical abstract:**

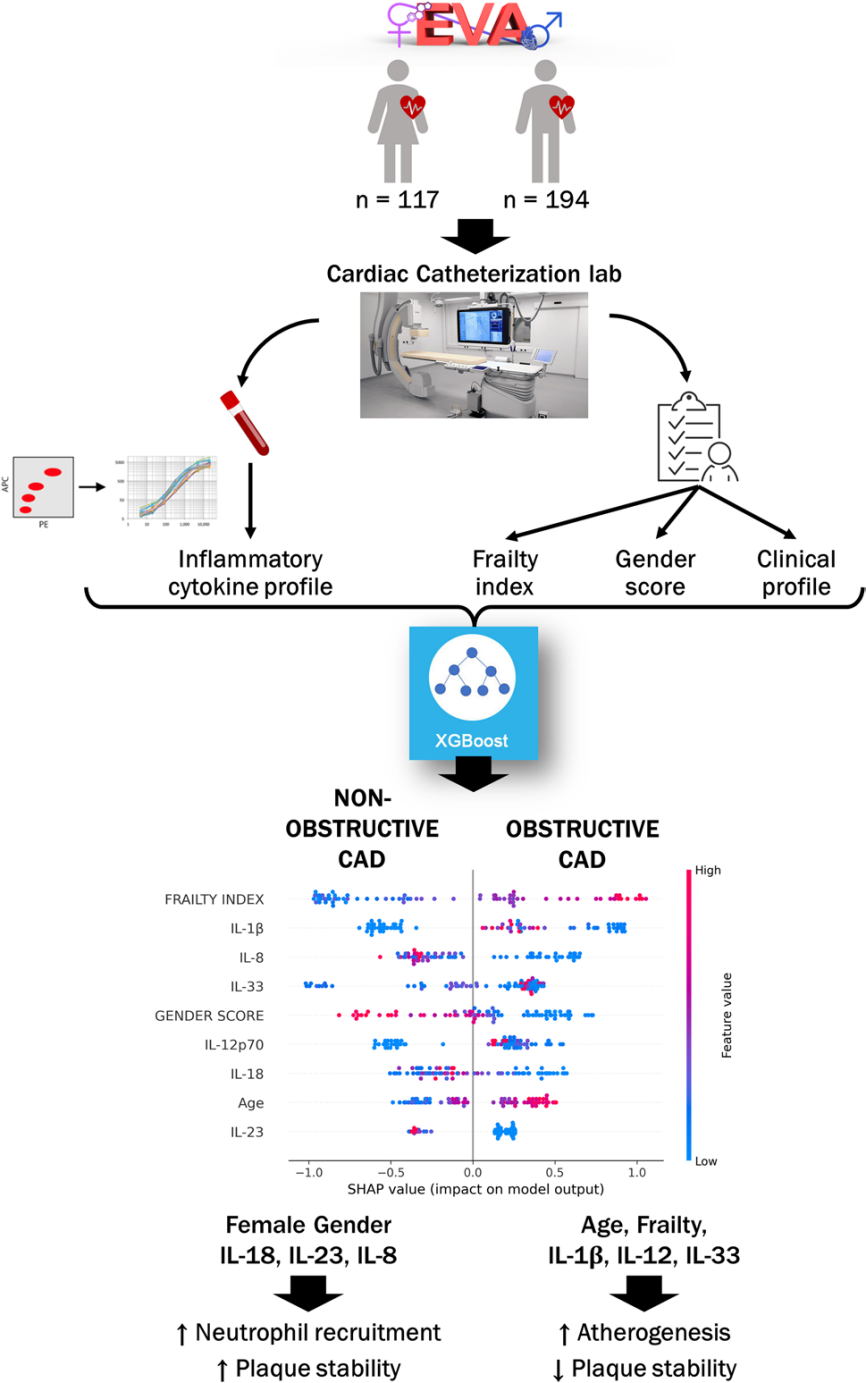

**Supplementary Information:**

The online version contains supplementary material available at 10.1007/s00392-023-02193-5.

## Introduction

Although adverse clinical outcomes from ischemic heart disease (IHD) have been on a linear decline over the last decade, the burden remains high in both females and males [1]. Heterogeneity in manifestations, prognosis, and response to treatments for IHD between males and females have been reported, with females less likely receiving high-quality cardiovascular care [2]. The reasons behind such disparities might stand on how biological sex and sociocultural gender (*i.e.,* identity, roles, relations, and institutionalized gender) interact in shaping the development and the progression of IHD [3]. The unmet need to foster the collection of sex- and gender-unbiased data to inform our understanding of diseases has surged to improve outcomes and personalize the management of IHD patients in the era of precision medicine [4]. In this respect, machine-learning (ML) techniques have boosted our ability to predict outcomes; nevertheless, to obtain fair and equitable algorithms, the wealth of features used for training the models should be representative of the biological, functional, and sociocultural complexity to overcome selection biases [5].

IHD is not anymore synonymous with obstructive flow-limiting coronary artery disease (CAD), especially in females that are more commonly affected by non-obstructive disease [6]. The absence of obstructive CAD (i.e., < 50% diameter reduction at invasive coronary angiography) suggests the need to improve imaging tests to identify the different endotypes of either chronic or acute ischaemia with non-obstructed coronary arteries [7]. Furthermore, the mechanisms of myocardial ischemia in patients with or without obstructive CAD, including the key role of inflammation pathways [8, 9], are still far to be fully elucidated. Targeting individual interleukins (IL) in patients with atherothrombotic residual risk despite evidence-based standard therapy, has achieved a lower effect than expected (e.g., only a 15% risk reduction of adverse events in patients treated with canakinumab, an IL-1β blocker) suggesting that other products of the inflammasome might be involved, such as IL-18 [10], and that individuals may have distinct cytokine signatures depending on the type of CAD. Of note, while the role of inflammation in atherosclerotic obstructive CAD is well established, little is known on the inflammatory burden associated with non-obstructive CAD. As females are less likely to develop atherosclerosis [11], it is biologically plausible that other thrombo-inflammatory mechanisms sustain the ischemia. To better the multifaced complexity of CAD phenotypes throught a sex- and gender-unbiased approach, we developed a ML-based model for the supervised prediction of the clinical, functional, biological, and psycho-social features associated with non-obstructive and obstructive CAD among a consecutive unselected cohort of adults hospitalized for IHD.

## Methods

### Study population

The “Endocrine Vascular disease Approach” (EVA) project (ClinicalTrials.gov identifier NCT02737982), is an observational, prospective study, aimed at exploring sex- and gender-related differences in the interaction between platelet function, sex hormone balance, and coronary microvascular dysfunction in IHD. Study design of EVA has been previously published [12]. Briefly, EVA is an observational registry of individuals (> 18 years), who were referred to the cardiac catheterization laboratory for undergoing conventional coronary angiography (CCA) and/or percutaneous coronary intervention for either acute coronary syndrome (ACS) or stable angina. In-patient and outpatients were recruited at the University Hospital Policlinico Umberto I of Rome, Italy, between April 2016 and March 2020. Based on angiography, we have classified IHD patients as follows: (1) ischemia with obstructive CAD, that is ≥ 50% diameter stenosis; and (2) ischemia with non-obstructive CAD, that is < 50% diameter stenosis [11]. A multidimensional approach was applied to collect a granularity of participants’ characteristics including clinical, laboratory and treatment variables. The study was conducted in full conformance with the principles of the Declaration of Helsinki, and it was approved by the local Ethics Committee of Policlinico Umberto I, Sapienza University of Rome, Rome, Italy (reference 3786, 24/09/2015). Written informed consent has been obtained from all patients.

### Calculation of the gender score

At enrolment, gender-related variables pertinent to the four domains gender encompasses (i.e., identity, roles, relations, institutionalized gender) [13] were collected. The GENESIS-PRAXY methodology provided the framework used to generate a composite measure of gender [14, 15] (Supplemental Material, Appendix A, Supplemental Table 1 and 2). Briefly, Principal component analysis (PCA) methodology was used to select the unique set of covariates that accounted for a cumulative variance of greater than 80% of the data. The optimized set of gender-related variables were used to create a multivariable logistic model with biological sex as the dependent variable and gender-derived components as covariates. A gender score was calculated through the construction of a propensity score that defined the conditional probability of being a female versus a male using gender-related variables. This score ranges from 0 to 100, with higher scores relating to characteristics traditionally ascribed to women. Among the EVA participants, the eight variables that were independently associated with biological sex and included in the EVA gender score (according to their own weight based on their coefficient estimate) were: (1) engagement in social leisure activities (including sports of moderate/strenuous intensity, dancing); (2) being married or living with a partner; (3) responsibility for housework; (4) housework hours; (5) being the primary earner of the household; (6) level of stress home; (7) emotional support received (i.e. level of emotional support); (8) trust and confidence (i.e. someone available that you can trust and confide in).

### Rockwood frailty index

Frailty was assessed according to a 50-item frailty index (FI) (Supplemental Material, Appendix B, Supplemental Table 3), built on the model designed by Rockwood and Mitnitsky [16, 17]. FI was computed based on a multidimensional evaluation that included patients’ comorbidities, baseline biomarkers, reported symptoms, level of autonomy in activities of daily living and perceived stress. Concomitant comorbidities were assessed at baseline during clinical interview, with each comorbidity considered as a possible single deficit. Haemoglobin, neutrophil/lymphocyte ratio, c-reactive protein, creatinine clearance according to CKD-EPI formula and plasmatic albumin were assessed as biomarkers. Each biomarker was categorized and scored as a single possible deficit, as reported in Supplementary Table 3. Autonomy of patients was assessed according to the Duke Activity Status Index (DASI) score [18], with each item considered as a single possible deficit. Chest pain at index event was assessed according to the Rose Angina Questionnaire [19], with each item considered as a single possible deficit. Patients’ perceived stress was evaluated according to the perceived stress scale (PSS-10) [20], with each item considered as a single possible deficit. Calculation of FI was performed as the ratio of the total deficit found for each patient over the total number of possible deficits examined. According to the usual clinical use, a FI ≥ 0.25 was considered as a cut-off to define presence of Frailty [21].

### Inflammatory cytokines profiling

The concentration of inflammatory cytokines was assessed in serum samples by multiplex bead-based flow cytometric assay (Biolegend, Inflammation Panel I, catalogue number 740809), according to the manufacturer instructions. Arterial blood was collected from the coronary circulation during the angiography and before PCI. Within 2 h from withdrawal, blood was centrifuged for 20 min at 2000 g and coded serum samples were stored at − 80 °C until batch analysis. According to a previous study, the arterial samples are suitable for testing biomarkers of platelet function and cytokines [22].

After thawing, the serum samples were immediately centrifuged at maximum speed and transferred to new tubes. A small volume of each serum (25 μl) was diluted 1:1 in Assay buffer provided in the kit. Each serum was incubated with 13 bead populations distinguished by size and internal APC fluorescent dye, which bind to 13 distinct human inflammatory cytokines and chemokines, including IL-1β, IFN-α2, IFN-γ, TNF-α, MCP-1 (CCL2), IL-6, IL-8 (CXCL8), IL-10, IL-12p70, IL-17A, IL-18, IL-23, and IL-33. The following day the beads were incubated first with cytokine-specific biotinylated antibodies and then with Streptavidin–phycoerythrin and immediately acquired at a BD Accuri C6 Plus flow cytometer. Cytokine-specific populations were segregated based on the size and internal APC fluorescence intensity. The concentration of a particular cytokine was quantified based on the PE fluorescent signal according to a standard curve generated in the same assay. Measurements were ascertained while blinded to the sample origin. All samples were assayed in duplicate, and those showing values above the standard curve were retested with appropriate dilutions.

### Statistical analysis

Univariate descriptive analysis of the baseline clinical, biological, functional, and psycho-social variables by type of CAD was performed. Normality for continuous variables has been assessed using the Kolmogorov–Smirnov test. Continuous data are represented as mean and standard deviation (SD) or median and interquartile range (IQR) (25th, 75th percentile) and compared with two-tailed *t* tests or Mann–Whitney tests, as appropriate. Categorial variables are presented as number of participants (percentage) and compared using chi-square test. Only *p* values < 0.05 are regarded as statistically significant. Analyses have been performed using Graph-Pad Prism 9.0 and SPSS v. 25.0 (IBM, NY, USA).

### Machine learning CAD classification model

**Fig. 1:**
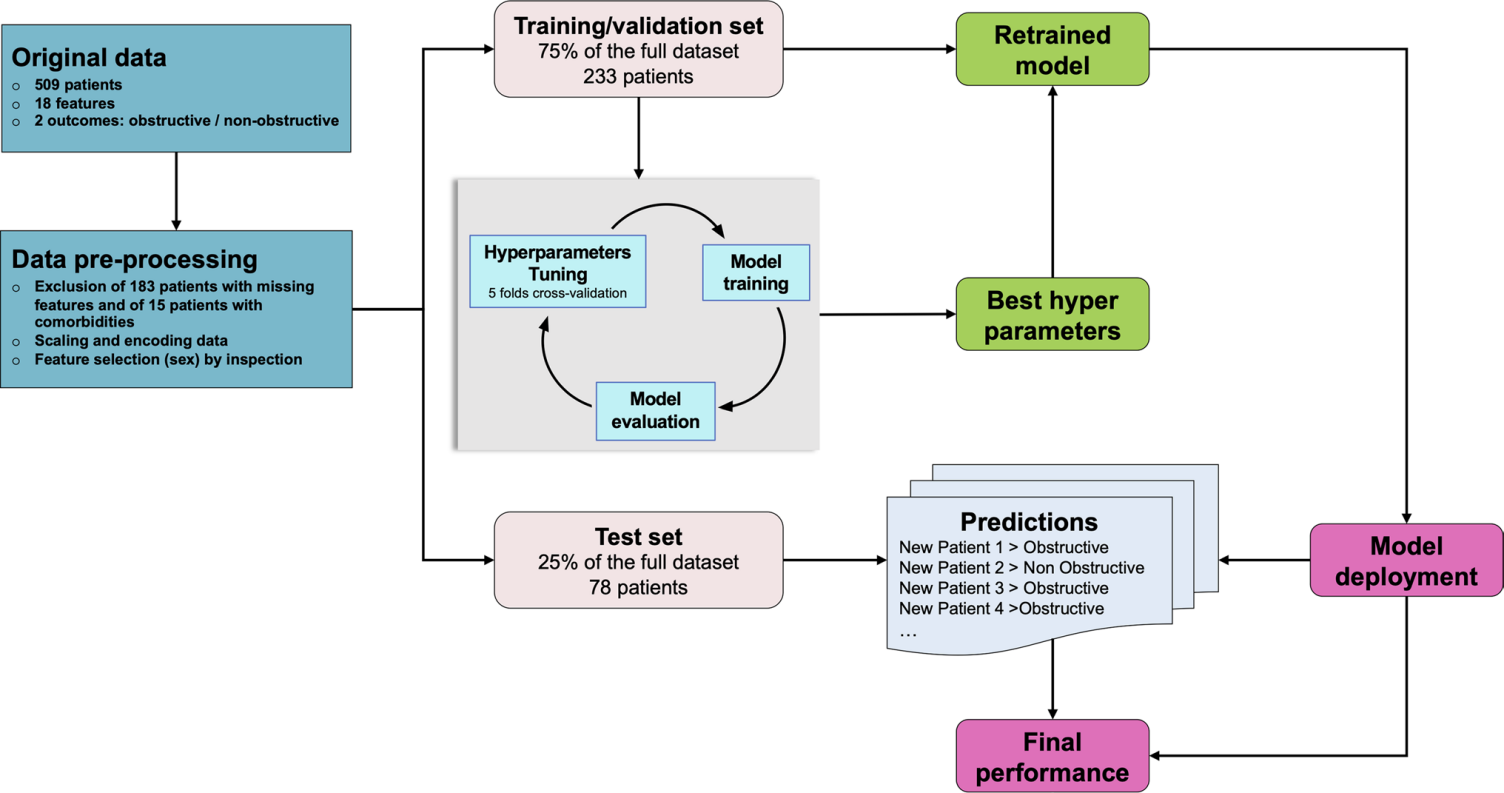
Definition of the machine-learning classification model. Schematic representation of the working process to define and optimize the ML-based model. After data pre-processing, 75% of the data is used to train an XGBoost model including the tuning of the hyperparameters by means of a fivefold cross-validation procedure. The hyperparameters that give the best average validation values of the key performance indicators are chosen and the model is retrained on the full training set with the optimal setting. Eventually, the model is deployed and used to predict the type of CAD of the patients in the test set (25% of data) and the final performance (accuracy and precision) of the model is determined

Briefly, the steps to define and optimize the ML-based model are summarized in Fig. 1.

### Data pre-processing and analysis

The initially available dataset consisted of 509 patients with 18 features (i.e., sex, gender score, FI, age, Body Mass Index [BMI], IL-1β, IFN-α2, IFN-γ, TNF-α, MCP1, IL-6, IL-8, IL-10, IL-12p70, IL-17A, IL-18, IL-23, IL-33).

Data were pre-processed to overcome quality issues due to limitations of measurement devices, human errors, or problems in the data collection process. In particular, 15 more patients were removed from the dataset by means of physicians’ expertise since the values of at least one feature were clearly incorrect resulting in a dataset of 494 patients. Over the 494 available patients, we eliminated those that had more than 12 features (over the 18) reporting a null value, because it makes no sense to impute more than 66% of the values. Among the remaining 339 patients, further 28 patients had at least one missing feature. We considered the possibility of imputing the missing features using different rules. However, this did not improve the results, as reported in the Supplemental Table 4, and we did not consider imputation in the final results.

As a first step, a linear correlation analysis was conducted, and the Pearson correlation matrix **(**Supplemental Fig. 1) was constructed to check the existence of linear correlation among features. Of note, we observed an expected high linear correlation between sex and gender score. Hence, we decided to keep the gender score in the model. In order to improve the performance and training stability of the model, the features it the dataset were standardized so they have a mean of zero and a variance of one.

As the final dataset (*n* = 311) turned out to be imbalanced, for a prevalence of patients with obstructive CAD (67.5%), we applied both undersampling or oversampling procedures, such as SMOTE (Synthetic Minority Over-sampling Technique) [23]; this procedure did not improve model performance on the dataset and resampling techniques performed less efficiently than the usage of the original imbalanced database. Thus, none of these techniques were used.

### Machine learning model choice, tuning, and testing

The 311 patients were used to train and test a supervised classification ML model (Fig. 1). Several classification models were tested (from Logistic Regression [LR] to more complex ML models such as Support Vector Machines, Random Forest [RF] and ensemble methods, etc.) in order to select the best model in extracting underlying patterns linking the bio-psycho-social features in input with the outcome of type of CAD.

Data were split so that 75% of the data were used as training-validation set while the remaining 25% was put aside and used as test set to check the quality of the model obtained, that is its capability to correctly predict the outcome of unseen patients. We provide in the supplementary material the Supplemental Table 5 with baselines of both training and test cohorts to show that there are no significant differences.

Each ML model relies on some hyperparameters that need to be set. Several configurations of hyperparameters were assessed to find the optimal set. In particular, a random-search hyperparameter tuning was applied and the best setting was selected based on best Key Performance Indicators (KPIs) evaluated on average using a fivefold cross-validation. The performance on the associated testing risk scores was reported too. The metrics used to assess the quality of the models and compare them, and the features selection process are specified in the online supplement (Supplemental Figs. 2, 3). Extreme gradient boosting (XGBoost) [24], already successfully applied in various medical applications [25], was the most performing prediction model. The Python version implemented in the library xgboost, version 1.2.1, was used.

### Shapley values and features of importance in the analysis

To provide an interpretable prediction model, we adopted the Tree SHAP (SHapley Additive exPlanations) [26] for tree-based machine-learning models [27, 28]. For each patient in the dataset, SHAP aims to compute, in principle, the contribution of each feature in making the model return a certain output and produces a graphical representation of the feature’s importance. In fact, each feature is shown on the vertical axes of a graph where the higher is the position (ranking), the more influential is the characteristic. In the graph for each feature (namely on the horizontal lines), the dots represent patients, and the colour indicates whether the value of the characteristic considered is high or low in relation to the range of values (red refers to high values and blue to low values). The graph has a median line and the farther the point is from the median line, the stronger is the influence on the output, with the points on the right correlating positively with obstructive CAD and the points on the left negatively, thus predicting the opposite outcome (non-obstructive CAD).

## Results

Among the overall EVA cohort (*n* = 509), 311 individuals (mean age 67 ± 11 years, 38% females; 67% obstructive CAD) with complete data on the features included in the ML model were analysed.

The baseline characteristics of the cohort by type of CAD are summarized in Table 1. Adults with non-obstructive CAD were more likely females, younger, with a lower BMI and burden of comorbidities as compared with individuals with obstructive CAD. The clinical presentation was less frequently an ACS, with a lower percentage of individuals with STEMI in non-obstructive CAD individuals and less prior history of IHD than those with obstructive CAD. Conversely, they had more likely a higher gender score (i.e., social characteristics traditionally ascribed to women), and a higher frailty index (i.e., low physiological reserve).

**Table 1:** Baseline features of the 311 individuals with ischemic heart disease stratified by type of CAD

	Overall cohort (*n* = 311)	Non-obstructive CAD (*n* = 101)	Obstructive CAD (*n* = 210)	*P* value
**Clinical variables**				
Age (years), median [IQR]	68.0 [60.0, 75.0]	65.0 [56.0, 73.0]	69.5 [61.3, 76.0]	0.002
Female sex, *n* (%)	117 (37.6)	48 (47.5)	69 (32.9)	0.018
BMI (median [IQR])	26.6 [24.2, 29.7]	25.9 [23.4, 28.8]	26.9 [24.7, 30.1]	0.029
Family Hx CVD, *n* (%)	193 (62.1)	61 (60.4)	132 (62.9)	0.769
Smoking, *n* (%)	76 (24.7)	25 (24.8)	51 (24.6)	1.000
Hypertension, *n* (%)	243 (78.1)	67 (66.3)	176 (83.8)	0.001
Heart failure, *n* (%)	35 (11.3)	9 (9.0)	26 (12.4)	0.492
Dyslipidaemia, *n* (%)	152 (49.0)	34 (33.7)	118 (56.5)	< 0.001
Type 2 diabetes, *n* (%)	82 (26.4)	16 (15.8)	66 (31.4)	0.005
Known IHD, *n* (%)	98 (31.5)	18 (17.8)	80 (38.1)	0.001
Prior AMI, *n* (%)	67 (21.5)	12 (11.9)	55 (26.2)	0.006
Vascular disease^§^, *n* (%)	77 (24.8)	11 (10.9)	66 (31.4)	< 0.001
Prior stroke/TIA, *n* (%)	32 (10.3)	9 (8.9)	23 (11.0)	0.722
Dementia, *n* (%)	0 (0)	0 (0)	0 (0)	NA
End-stage chronic kidney/dialysis, *n* (%)	4 (1.3)	2 (2.0)	2 (1.0)	0.829
COPD, *n* (%)	33 (10.6)	13 (12.9)	20 (9.5)	0.483
Acute coronary syndrome, *n* (%)	165 (53.1)	41 (40.6)	124 (59.0)	0.003
STEMI, *n* (%)	24 (7.7)	2 (2.0)	22 (10.5)	0.016
**Gender-related variables**				
Gender score (median [IQR])	0.22 [0.06, 0.62]	0.37 [0.09, 0.71]	0.17 [0.05, 0.57]	0.009
Primary responsibility for doing housework, *n* (%)	129 (41.5)	56 (55.4)	73 (34.8)	0.001
Being married or living with partner, *n* (%)	215 (69.1)	59 (58.4)	156 (74.3)	0.007
Primary earner status, *n* (%)	200 (64.3)	64 (63.4)	136 (64.8)	0.909
Engagement in recreational social activities, *n* (%)	165 (53.1)	58 (57.4)	107 (51.0)	0.342
Emotional support received (range 1–5) (median [IQR])	4 [3, 5]	4 [4, 5]	4 [3, 5]	0.182
Number of hours spent for household chores (median [IQR])	6 [2, 14]	7 [2, 15]	6 [2, 14]	0.340
Trust and Confidence in someone (range 1–5) (median [IQR])	4 [4, 5]	4 [4, 5]	4 [4, 5]	0.091
Level of stress at home (range 1–10) (median [IQR])	2 [1, 5]	3 [1, 5]	2 [1, 4]	0.025
**Physiological reserve**				
Frailty index (median [IQR])	0.31 [0.22, 0.39]	0.25 [0.19, 0.34]	0.33 [0.25, 0.42]	< 0.001
Number of available items of frailty index (range 0–50) (median [IQR])	48 [39.5, 49]	48 [40, 49]	40 [39, 49]	0.009
Percentage of available items (%)				0.006
‒50%	2 (0.6)	0 (0.0)	2 (1.0)	
‒70–79%	76 (24.4)	14 (13.9)	62 (29.5)	
‒80–100%	233 (74.9)	87 (86.1)	146 (69.5)	

*BMI* body mass index; *IQR* interquartile range; *IHD* ischemic heart disease; *AMI* acute myocardial infarction; *CAD* coronary artery disease; *COPD* chronic obstructive pulmonary disease; *DAPT* dual antiplatelet therapy; *Hx* history; *PAD TIA*, transient ischemic attack; *SD* standard deviation; *STEMI* ST elevation myocardial infarction, *PCI* percutaneous coronary intervention

^§^Peripheral artery disease and/or Abdominal Aortic Aneurysm, and/or Carotid Stenosis

### Individuals with non-obstructive CAD have a distinct cytokine signature versus those with obstructive CAD

**Fig. 2:**
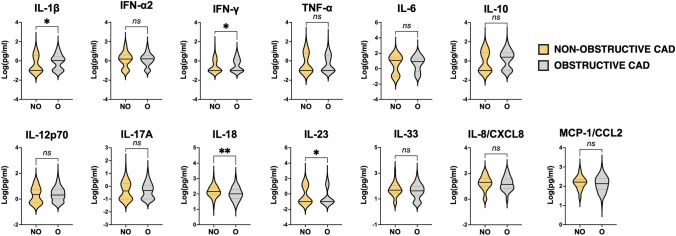
Cytokine profile of obstructive and non-obstructive CAD patients. Violin plots display in logarithmic scale the concentration (pg/ml) of 13 cytokines measured by multiplex bead-based flow cytometric assay in coronary serum samples of non-obstructive (NO; < 50% diameter stenosis) or obstructive (O; ≥ 50% diameter stenosis) CAD patients. A straight line indicates the median and the dotted lines show the interquartile range (IQR) (25th, 75th percentile). The significance of differences between median values was tested by Mann–Whitney tests for independent samples (*p ≤ 0.05, **p ≤ 0.01)

We measured the concentration of 13 cytokines/chemokines in coronary serum samples collected at T0. On average, we detected elevated levels of IL-6 in both obstructive and non-obstructive CAD patients (median [IQR]: 12.2 [0.0–31.4] pg/ml in non-obstructive CAD; 8.5 [0.4–23.5] pg/ml in obstructive CAD; *p* = 0.96) suggesting that both conditions are associated to a state of low-grade inflammation (Fig. 2).

**Fig. 3:**
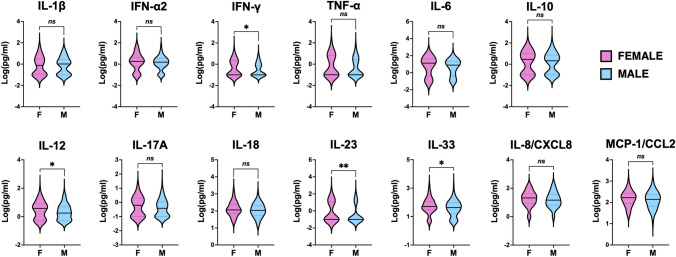
Cytokine profile of female and male individuals with CAD. Violin plots display in logarithmic scale the concentration (pg/ml) of 13 cytokines measured by multiplex bead-based flow cytometric assay in coronary serum samples of female (F) and male (M) CAD patients. A straight line indicates the median and the dotted lines show the interquartile range (IQR) (25th, 75th percentile). The significance of differences between median values was tested by Mann–Whitney tests for independent samples (*p ≤ 0.05, **p ≤ 0.01)

The same pattern was shared between the sexes, however, female patients had a higher inflammatory burden (Fig. 3), as they displayed significantly higher levels of IFN-γ (median [IQR]: 0.1 [0.0–2.2] pg/ml in females; 0.0 [0.0–0.8] pg/ml males; *p* = 0.0381), IL-12 (median [IQR]: 3.8 [0.5–7.7] pg/ml in females; 1.8 [0.5–4.9] pg/ml males; *p* = 0.0455), IL-23 (median [IQR]: 0.0 [0.0–13.4] pg/ml in females; 0.0 [0.0–0.0] pg/ml males; *p* = 0.0455) and IL-33 (median [IQR]: 50.8 [29.3–118.5] pg/ml in females; 42.7 [16.7–91.5] pg/ml males; *p* = 0.0455), compared to male patients.

**Fig. 4:**
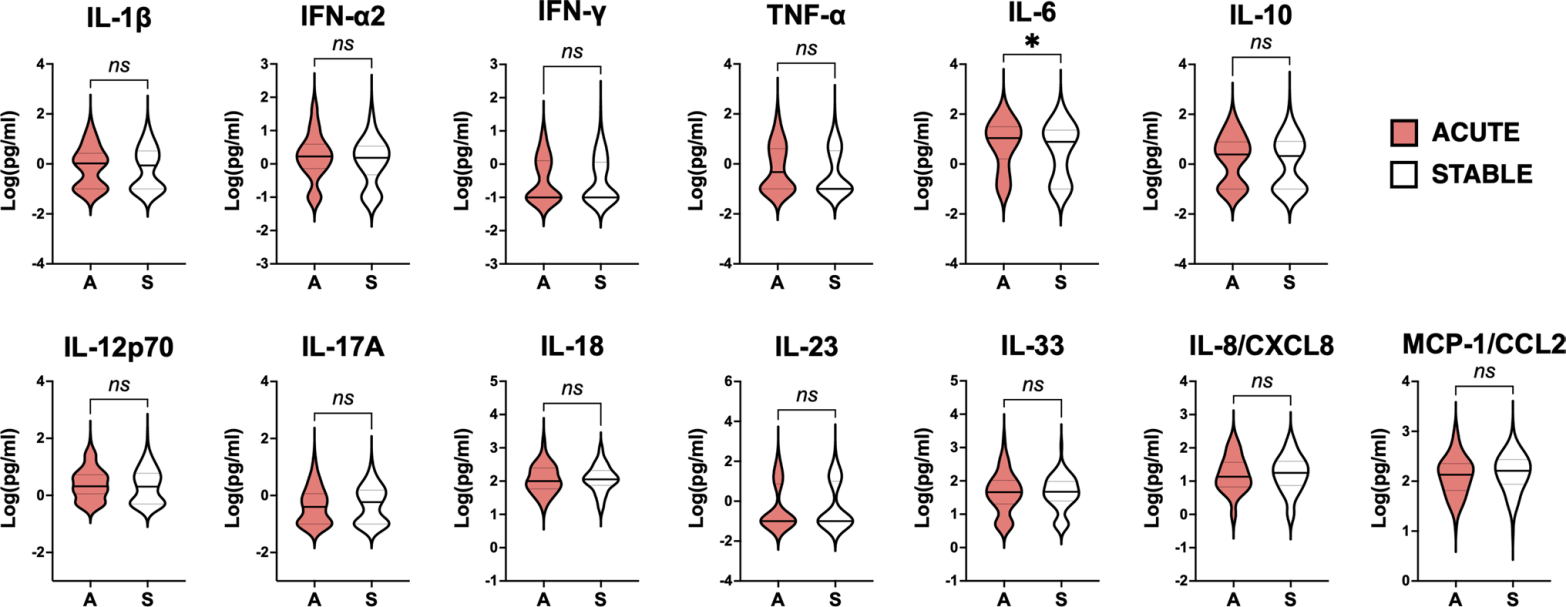
Cytokine profile of patients with acute and stable CAD. Violin plots display in logarithmic scale the concentration (pg/ml) of 13 cytokines measured by multiplex bead-based flow cytometric assay in coronary serum samples of CAD patients with acute (A) or stable (S) presentation. A straight line indicates the median and the dotted lines show the interquartile range (IQR) (25th, 75th percentile). The significance of differences between median values was tested by Mann–Whitney tests for independent samples (*p ≤ 0.05)

Elevated concentration of IL-6 (median [IQR]: 7.9 [0.0–23.2] pg/ml in stable CAD; 11.1 [1.7–31.4] pg/ml in acute CAD; *p* = 0.048) was the only feature that significantly discriminated those individuals presenting with ACS (Fig. 4). Conversely, we detected several differences in the cytokine serum concentration between obstructive and non-obstructive CAD patients. Specifically, we found significantly higher levels of IL-18 (median [IQR]: 142.7 [87.65–247.8] pg/ml in non-obstructive CAD; 101.1 [51.41–201.1] pg/ml in obstructive CAD; *p* = 0.0033) and IL-23 (median [IQR]: 0.0 [0.0–10.87] pg/ml in non-obstructive CAD; 0.0 [0.0–5.72] pg/ml in obstructive CAD; *p* = 0.0384) in patients with non-obstructive CAD, and higher concentrations of IL-1β (median [IQR]: 0.0 [0.0–3.47] pg/ml in non-obstructive CAD; 1.07 [0.0–2.69] pg/ml in obstructive CAD; *p* = 0.0208) and IFN-γ (median [IQR]: 0.0 [0.0–0.83] pg/ml in non-obstructive CAD; 0.0 [0.0–1.38] pg/ml in obstructive CAD; *p* = 0.0469) in patients with obstructive CAD.

### ML-based model for the prediction of non-obstructive and obstructive coronary artery disease

We initially applied the XGBoost method in connection with the SHAP tools to identify clusters of cytokines that associate with non-obstructive (negative SHAP value) or obstructive (positive SHAP value) CAD. As a first analysis, we trained a prediction model, only providing cytokines data as features and we observed that obstructive CAD associates with high concentrations of IL-1β, IL-12, IL-33, IL-10, IFN-γ, and MCP-1, while non-obstructive CAD is more likely to associate with high levels of IL-23, IFN-α2, IL-18, IL-8, IL-6, TNF-α and IL-17a. However, the model including only biological data achieved a modest prediction accuracy (70.5%) and precision (75.9%) (Supplemental Fig. 4).

To pursue a more holistic representation of adults with CAD, we included the following features: age, gender score and frailty index. Introducing these composite measures of individuals’ complexity into the XGBoost model substantially improved the accuracy (79.5%) and precision (83.6%) (Supplemental Fig. 5).

Finally, to optimize the model that would discriminate obstructive from non-obstructive CAD with the minimal amount of information, we excluded features that appeared to be less influential in discriminating CAD according to the SHAP plot and that were redundant based on established relationships between cytokines. Namely, we did not include those features that were lower along the vertical axis (i.e., less important in discriminating CAD types) and those that distributed near the mid-line of the SHAP plot (i.e., correlated poorly with either one of the CAD types). For example, IL-17A was the lowest ranking feature both in the SHAP plot with cytokines alone and in the full features SHAP graph; further, it clustered around the mid-line, thus having a low Shapley value. In many inflammatory states, IL-17A correlates with IL-23, that is the main cytokine regulating IL-17-producing T helper cells (Th17). Being upstream, IL-23 is likely to control more signalling pathways than IL-17A, and therefore, to provide more biological information. Thus, IL-17A was selected out. Similarly, we excluded IFN-γ that is produced by type 1T helper cells (Th1) downstream of IL-12 stimulation.

**Fig. 5:**
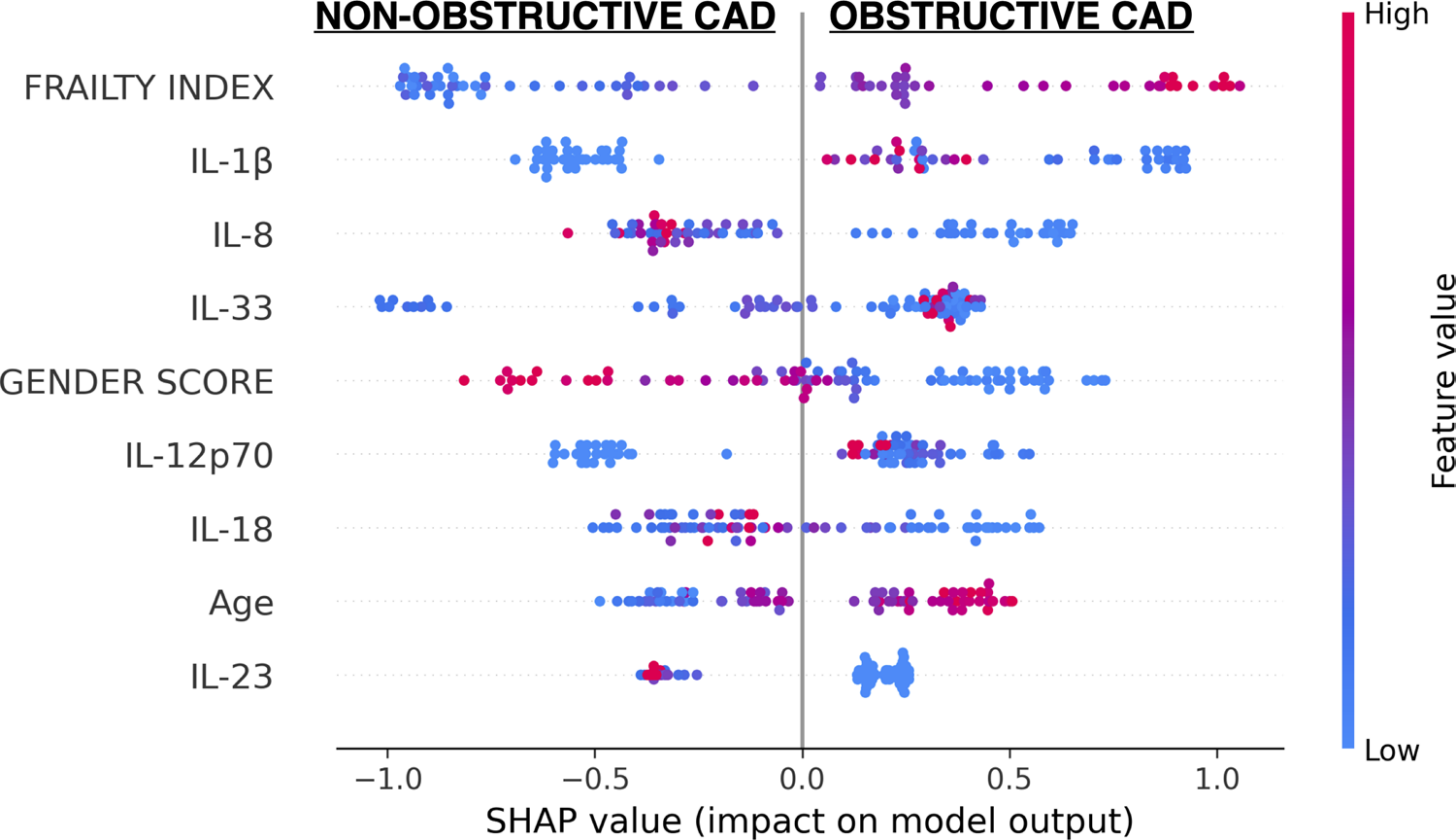
Machine-learning model that integrates biological, clinical, functional and psycho-social features to predict obstructive and non-obstructive CAD. SHAP Plot of the best performing ML-based model. Each feature is shown on the vertical axes of a graph where the higher is the position (ranking), the more influential is the characteristic. In the graph for each feature (namely on the horizontal lines) the dots represent patients, and the colour indicates whether the value of the characteristic considered is high or low in relation to the range of values (red refers to high values and blue to low values). The graph has a median line and the farther the point is from the median line, the stronger is the influence on the output, with the points on the right correlating positively with obstructive CAD and the points on the left negatively, thus predicting the opposite outcome (non-obstructive CAD)

We also excluded those cytokines that despite having a relatively high ranking in the SHAP plot had average concentrations comparable to healthy subjects, such as IFN-α2, or that substantially reduced the accuracy and the precision of the model, as for the anti-inflammatory cytokine IL-10. The full list of the trials is reported in Supplemental Table 6. Ultimately, we achieved (see quality assessment in methods section) the final model that includes nine features (i.e., gender score, age, frailty index, IL-1β, IL-18, IL-8, IL-23, IL-12p70, IL-33) and that could discriminate between obstructive and non-obstructive CAD with 83% accuracy and 87% precision (Fig. 5).

Furthermore, we tested the effect of adding IL-6, the most widely used cytokine biomarker of IHD, in the final model but this resulted in a reduction of the accuracy (79.5%) and the precision (84.9%) (Supplemental Fig. 6).

In Supplemental Tables 7 and 8, we compared the performance of the chosen XGBoost method versus RF or a LR model using the nine features selected and the ROC curve as a KPI. Thus, we  proved that the chosen method outperforms more traditional statistical learning models (Supplemental Fig. 7). We also tested if a model including only frailty, age, and gender, without the biological variables, would have been fair but the results decreased both in accuracy (70.4%) and precision (71.4%) (Supplemental Table 9).

Finally, we used the selected nine features to construct two separate models for patients presenting with acute or stable CAD, respectively. Interestingly, we found that the correlations between CAD type and high cytokine concentrations were almost unchanged when considering patients with acute presentation as compared with the entire cohort (Supplemental Fig. 8). Conversely, among stable CAD patients, high concentrations of all cytokines except IL-33 were associated with obstructive CAD.

## Discussion

In the present study, we developed an interpretable ML-based model that discriminates non-obstructive and obstructive CAD through features that capture (i) the reduction of physiological functional reserve of individuals (i.e., frailty); (ii) the inflammatory burden of IHD; and (iii) the complexity that gender encompasses (identity, role, relations, and institutionalized gender). With a minimal number of nine variables, we have optimized a predictive model with 83% accuracy and 87% precision. In addition, we have gained insights on the complexity behind CAD. Some features spanning from biological to functional and gendered psycho-social factors can help to envision and test in further studies which of the input features were most influential in discriminating the two distinct types of CAD. We found that while obstructive CAD associates with increased frailty index, older age and a cytokine signature characterized by IL-1β, IL-12p70 and IL-33, non-obstructive CAD is more likely to associate with a higher gender score and with a cytokine signature characterized by IL-18, IL-8, IL-23.

Despite advancements in therapeutics, CVD persists to be the leading cause of morbidity and mortality in both males and females worldwide. Nevertheless, sex differences have been reported in the clinical phenotype and trajectory of IHD [6]. Indeed, IHD is a multifactorial and complex condition, that requires a multidimensional approach to better inform our understanding of myocardial ischemia. Non-obstructive coronary atherosclerosis is becoming a frequent finding in individuals with myocardial ischemia [29], especially females. Beyond biological sex, gender may play a role as well. Frequently, sex differences in disease and outcomes can be explained due to the gendered distribution of factors between men and women [30]. Although mechanisms of action are largely unclear, it is suggested that gender-related factors can further exacerbate the detrimental effect of established risk factors of CVD [30]. Commonly, the wealth of gender-related factors is not collected in clinical studies with a consequential lack of testing the effect of gender on outcomes. Conversely, in the EVA study, variables capturing the four domains that gender encompasses where available. In the EVA project grounded on the prior work of the GENESIS-PRAXY experience [14, 15], we collected a wealth of gender-related variables and were able to construct the gender score (as reported in supplementary material) that defines the probability of being a man or a woman, based on the gendered features of the individuals. Therefore, the inclusion of the gender score in the modelling provides a more balanced and representative measure of the intersection between sex and gender in shaping coronary health. Moreover, the inclusion in the model of both would have generated collinearity issues for the methodology used to construct the score. Interestingly, in our model, the feminine gender score was a feature strongly associated with non-obstructive CAD suggesting that bio-psycho-social characteristics are modifiers of cardiovascular health.

In the last few years, the concept of frailty, originally conceived in the geriatric medicine field, gathered a lot of attention from the general medical audience. Frailty is considered as a medical syndrome characterized by a diminished strength, endurance and reduced physiological function that increases subjects’ vulnerability and entails and increased risk for disability and death [31, 32]. Application of the cumulative deficits model to measure frailty helps in performing a comprehensive evaluation of subjects’ physiological function impairment [16, 17] and provides a framework for the evaluation of biological ageing, as opposed to chronological ageing [33].

Even in the context of cardiovascular diseases, presence of frailty, irrespective of how it is measured, can discriminate those subjects at higher risk of adverse events, identifying more compromised patients [34, 35].

Moreover, translational research highlighted that there is a close relationship between a systemic inflammatory status, the ‘inflammageing’, and the presence of frailty [36]. According to that data, this chronic inflammatory status is highly associated with several components of the subjects’ physiological function (prevalent comorbidities, reduced muscle mass and strength, reduced mobility and impaired cognitive function). This entails a strong relationship between the inflammatory status and the presence of frailty. In this context, our data document how the level of frailty can directly affect the risk of developing a more severe coronary disease, in relation with a pro-inflammatory set of cytokines and a deprived social condition.

**Fig. 6:**
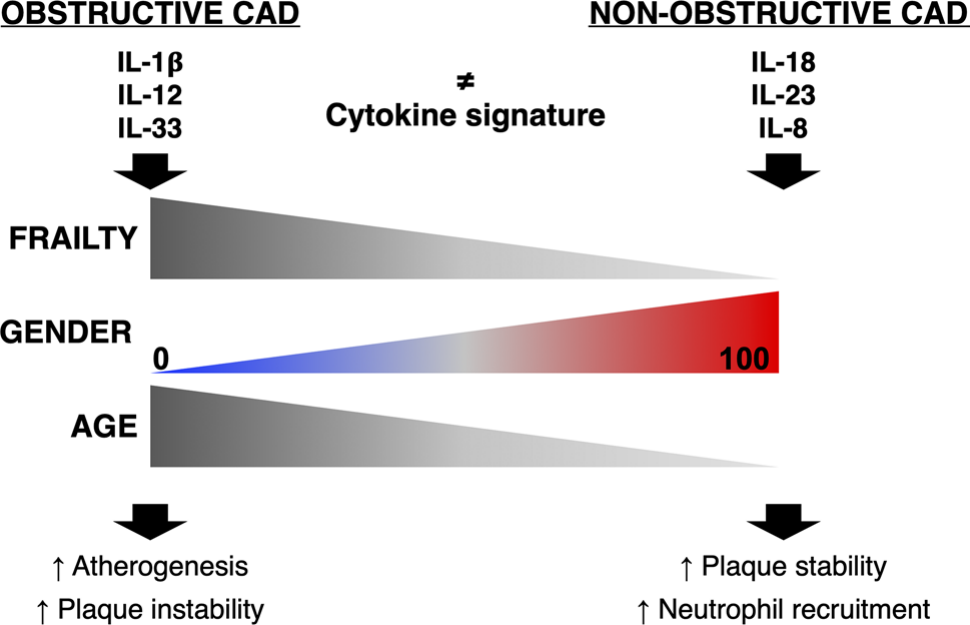
Conceptual framework of the study. Our ML-based model supports the idea that different inflammatory mechanisms underlie different type of CAD. We found that obstructive CAD was associated with increased frailty index, older age and a cytokine signature characterized by IL-1β, IL-12p70 and IL-33, which is pro-atherogenic and promotes plaque instability. Non-obstructive CAD was associated with a higher gender score (i.e., social characteristics traditionally ascribed to women) and with a cytokine signature characterized by IL-18, IL-8 and IL-23, which supports plaque stability and neutrophil recruitment

We are currently conducting mechanistic studies to understand the biological basis of these differences. Based on the data presented here, our working hypothesis is that different inflammatory responses are implicated in different types of CAD (Fig. 6, Graphical abstract). The cytokines included in our final predictive model do not reflect the full spectrum of inflammatory cytokines in the plasma of CAD patients, but only the few that discriminate between obstructive and non-obstructive CAD, independently from the patient clinical presentation (acute/stable). Interestingly, although IL-6 is the most studied inflammatory biomarker in IHD and we and others find it to be higher in acute presentations [37–40], it is not included in the final model as it is not informative to discriminate between obstructive and non-obstructive CAD, possibly because it participates to the pathogenesis of both types of CAD. Our results indicate the most important cytokines identifying obstructive CAD are IL-1β, which was indeed chosen as the target of the first anti-inflammatory trial to treat atherosclerosis [8] and IL-12, that drives the CD4 + Th1 response, which is pro-atherogenic [41]. The cytokine signature of non-obstructive CAD is characterized by IL-18, IL-8 and IL-23. The combination of IL-18 and IL-23 can act synergistically to induce CD4 + Th17 cells and γδT cells to produce IL-17a [42]. The function of the IL-23/IL-17 signalling axis is controversial in atherosclerosis [43] because, on one hand, it is atheroprotective by promoting collagen deposition and the formation of a thick fibrous cap that ensures plaque stability [44]. On the other hand, it promotes granulopoiesis [45] and, in synergy with TNF-α, induces the recruitment of neutrophils [46], which aggravate the inflammatory state. IL-18 has also been shown to stimulate endothelial cells to release IL-8 [47] (chemokine that recruits neutrophils), to expose adhesion molecules that facilitate the binding of platelets and neutrophils, and to induce endothelial cell apoptosis [48], which could contribute to plaque erosion. Thus, while in obstructive CAD, the inflammatory response drives atherosclerosis and plaque rupture, in non-obstructive CAD, the inflammatory response induces neutrophil engagement, which could support thrombus formation even in the absence of an unstable plaque through mechanisms including the release of microparticles [49, 50] and/or neutrophil extracellular traps [51].

These observations support the idea that targeting inflammation to reduce the risk of CVD could be a more successful strategy if we would target specific inflammatory pathways in a specific subset of patients. Subsequent analysis of the Canakinumab Anti-inflammatory Thrombosis Outcome Study revealed that inhibiting IL-1β does not modify the plasmatic levels of IL-18 [10]. Based on our data, IL-18 is more relevant than IL-1β in the pathogenesis of non-obstructive CAD, thus canakinumab may not protect from this type of CAD. Broader approaches like the NLRP3 inflammasome inhibitors could be effective in protecting a larger population but could pose more risks. A more successful approach could be to target a specific cytokine in the subjects more at risk of one or the other CAD. In this study, we move one step forward in identifying potential precise targets. While canakinumab or IFN-γ inhibitors could be used to target specifically subjects at risk of obstructive CAD, therapeutics targeting IL-23/IL-17, developed to treat psoriasis, could be used to prevent specifically non-obstructive CAD [52].

The subjects that could benefit the most from this precision medicine approach would be women. In our study, we show that females with CAD have a greater inflammatory burden than males, confirming the generally accepted notion that female mount stronger immune responses than men [53] and suggesting that this predisposition might put them at higher risk of CAD with inflammatory pathogenesis.

Finally, our manuscript has significant implications from a clinical point of view. Indeed, the evidence that the complex interaction between biological age, inflammatory burden and psycho-social factors (as encompassed by the gender score) increases the risk of developing obstructive CAD, advocate the need for more integrated care strategies, which could comprehensively handle patient care with a holistic care approach. Embracing complexity and developing approaches that accounted for it are already strongly advised in the field of cardiovascular diseases [54] and nowadays represents one of the standpoints in treating patients with multiple chronic conditions, as recommended by the WHO [55].

### Strengths and limitations

Several strengths of the study should be considered in the interpretation of the findings. We first provided an interpretable prediction tool for characterizing the phenotype of individuals with CAD through a multidimensional approach which captured the intersectionality between biology, physiological function, and sociocultural gender in shaping coronary vascular health.

We compared for the first time the inflammatory burden of obstructive and non-obstructive CAD. Even though we used a limited cytokines panel that does not cover the full spectrum of cytokines and chemokines implicated in the pathogenesis of CAD, we have moved one step toward identifying a distinctive signature for the two types of CAD and we are using this initial information to perform in depth mechanistic studies. The use of the SHAP tools delivers the possibility of explaining the role of the features within the black box ML-based model. The quantitative ranking obtained by the integrated use of ML and SHAP tools together with the expert-driven features selection, represents an effective way of producing interpretable explanations of the biological mechanism and the role of holistic factors in identifying obstructive/non-obstructive CAD. Moreover, the EVA study collected a broad wealth of gender-based variables that capture all the domains gender encompasses; therefore, we could generate a composite measure of gender and include it as distinctive feature of vascular health. We believe that our multidimensional model, taking together the mechanistic biological evaluation with the analysis of residual physiological function and gender vulnerability, provides a comprehensive and unique way to evaluate and interpret the presence of coronary artery disease.

The present findings should be interpreted in the light of several limitations.

The major limitation of this study is the small size of our cohort since ML approaches perform best with large datasets. As recently reported in a meta-analysis on ML-based studies on the prediction of cardiovascular disease [56], this issue commonly occurs depending on study design (e.g., single versus multi-center study), tools applied for defining cardiovascular disease (clinical vs advanced diagnostics). Nevertheless, due to the granularity and the multidimensional nature of the individual patient data in EVA, the study adds a different outlook in the prediction of CAD. Despite this limitation using ML techniques, we verified the low performance of traditional statistical approaches as compared with the ML-based selected. Thus, we could identify features that associate with either obstructive or non-obstructive CAD, which had not been identified by standard statistical techniques (Fig. 2). To assess the effectiveness of this approach, data from an independent cohort with larger sample size should be collected to make external validation of our model.

We defined non-obstructive CAD anatomically as < 50% diameter stenosis without performing functional tests, therefore, we included in non-obstructive CAD ischemic conditions that might have a different pathogenesis of ischemia especially in women [57, 58]; moreover, the anatomical stenosis based on minimal luminal area correlates poorly with the presence (or absence) of functionally obstructive disease [59].

The type of registry (i.e., patients who underwent CCA) might determine a selection bias effect on the composition of the EVA cohort as it is known that there are sex-based disparities in the accessibility to CCA [6]. Moreover, we might have missed some confounders due to the observational design of EVA. The generalizability might be limited as it is a single centre-based study.

Finally, despite identifying association does not imply a cause–effect mechanism, it generates a new hypothesis to be tested in further mechanistic studies.

## Conclusions

Integrating clinical, biological, and psycho-social features, we have optimized a sex—and gender-unbiased model that discriminates obstructive and non-obstructive CAD. Further mechanistic studies will shed light on the biological plausibility of the observed associations.

## Supplementary Information

Below is the link to the electronic supplementary material.

Supplementary file1 (DOCX 943 KB)

## Data Availability

The data underlying this article will be shared on reasonable request to the corresponding author.
