# Supplementary material for: A machine-learning based bio-psycho-social model for the prediction of non-obstructive and obstructive coronary artery disease
Source: Clin Res Cardiol. 2023 Apr 1;112(9):1263–77. doi: 10.1007/s00392-023-02193-5 (PMC10449670; doi:10.1007/s00392-023-02193-5)

**SUPPLEMENTAL MATERIALS**

**.**

Valeria Raparelli, Giulio Francesco Romiti, Giulia Di Teodoro, Ruggiero Seccia, Gaetano Tanzilli, Nicola Viceconte, Ramona Marrapodi, Davide Flego, Bernadette Corica, Roberto Cangemi, Louise Pilote, Stefania Basili, Marco Proietti, Laura Palagi, Lucia Stefanini, and EVA Investigators. A Machine-learning Based Bio-psycho-social Model for the Prediction of Non-obstructive and Obstructive Coronary Artery Disease.

**Appendix A - Composite Measure of Gender**

We performed a de-novo Principal Component Analysis and logistic regression starting with *all the 45 gender-related factors collected in EVA,* using biological sex as the dependent variable.

The EVA study includes at baseline the collection of 45 gender-related variables through self-administered questionnaires.

**Handling missing data:** before starting the PCA we checked for missing data and we decided to impute the missing data among the original cohort of 509 participants. We applied Simple random imputation (SRI) or hot deck imputation (Little & Rubin, 2002), as SRI is fit for random imputation of a single variable.

**PCA and Logistic regression:** Briefly, a composite measure of the gender-related characteristics was created, whereby the previously named variables were included in a principal component analysis. To determine which variables were gender-related in our cohort, the 33 variables were identified on the retained components from the principal component analysis out of the 45 available (**Supplemental Table 1)**.

Among the EVA participants, the 8 variables that were independently associated with biological sex and included in the EVA gender score (according to their own weight based on their coefficient estimate) were: 1) Engagement in Social Leisure Activities (including sports of moderate/strenuous intensity, dancing); 2) Being married or living with a partner; 3) Responsibility for housework; 4) Housework hours; 5) Being the primary earner of the household; 6) Level of Stress Home; 7) Emotional Support received (i.e. Level of Emotional Support); 8) Trust and Confidence (i.e. Someone available that you can trust and confide in) (**Supplemental Table 2**).

We quantified the predictive accuracy of the retained gender-related variables by using the c statistic to test the hypothesis that these variables were performing significantly better than chance to predict biological sex (indicated by a c statistic of 0.5). The final regression model including eight gender-related variables yielded a c statistic of 0.88, indicating that our model explained biological sex significantly better than chance. The choice of sex as dependent variable was made because to date, gender-related characteristics have mostly been defined historically based on social norms and expectations typically ascribed to men and women. These norms and expectations are likely to evolve with time, and to differ between sub-populations. We therefore aimed to define objectively the variables that were associated with the reality of being biologically female or male in the EVA cohort.

**Supplemental Table 1. Factor loadings from the principal component analysis related to each gender-related variable in the EVA dataset**

|  | **Factor1** |  | **Factor2** |  | **Factor3** |  | **Factor4** |  | **Factor5** |  | **Factor6** |  | **Factor7** |  | **Factor8** |  | **Factor9** |  | **Factor10** |  |
| --- | --- | --- | --- | --- | --- | --- | --- | --- | --- | --- | --- | --- | --- | --- | --- | --- | --- | --- | --- | --- |
| **AWARENESS_OF_SELFCARE** | -5 |  | -11 |  | -5 |  | -11 |  | -8 |  | -24 |  | 18 |  | 4 |  | 55 | * | 23 |  |
| **CALM_FEELING** | -5 |  | 61 | * | -26 |  | -16 |  | 0 |  | 1 |  | 4 |  | -10 |  | -1 |  | 5 |  |
| **CAREGIVER_STATUS** | -3 |  | -16 |  | -4 |  | -2 |  | -6 |  | -4 |  | 33 |  | -64 | * | -16 |  | 15 |  |
| **EDUCATION** | 6 |  | 4 |  | -9 |  | -7 |  | 10 |  | 20 |  | 7 |  | 12 |  | 64 | * | -18 |  |
| **EMOTIONAL_DYSFUNCTION** | 12 |  | -26 |  | 60 | * | 20 |  | -7 |  | -6 |  | -11 |  | 9 |  | 3 |  | -11 |  |
| **EMOTION_INTELIGENCE** | -19 |  | 3 |  | 12 |  | 7 |  | -2 |  | 20 |  | 3 |  | -36 |  | 47 | * | 11 |  |
| **ESSI_EMOTIONAL_SUPPORT** | 92 | * | 2 |  | 3 |  | 7 |  | 0 |  | -4 |  | 0 |  | -1 |  | 2 |  | 7 |  |
| **ESSI_GIVE_ADVICE** | 92 | * | -1 |  | 0 |  | 0 |  | 5 |  | 0 |  | 0 |  | -2 |  | -4 |  | -1 |  |
| **ESSI_HOUSEWORKS_HOURS_WEEK** | 5 |  | 6 |  | 5 |  | 3 |  | 75 | * | -4 |  | 4 |  | 22 |  | 9 |  | 9 |  |
| **ESSI_HOUSEWORKS_PERSON_RESP** | -3 |  | -12 |  | 2 |  | -1 |  | -82 | * | 1 |  | -13 |  | 7 |  | 1 |  | -2 |  |
| **ESSI_LOVEAFFECTION** | 85 | * | -5 |  | 6 |  | 9 |  | 4 |  | -6 |  | -5 |  | 7 |  | -2 |  | -1 |  |
| **ESSI_NEEDTOTALK** | 88 | * | -4 |  | 1 |  | 1 |  | 3 |  | -6 |  | -2 |  | -1 |  | -2 |  | -2 |  |
| **ESSI_TRUST_CONFIDE** | 91 | * | 2 |  | 1 |  | 7 |  | -1 |  | -2 |  | -2 |  | 3 |  | -1 |  | 3 |  |
| **INDEPENDENT_ATTITUDE** | -10 |  | -1 |  | 1 |  | -4 |  | -14 |  | 65 | * | 1 |  | -1 |  | 6 |  | -3 |  |
| **LEISURE_ACTIVITIES_SOCIAL** | 0 |  | 8 |  | -4 |  | -6 |  | -4 |  | 5 |  | 78 | * | -1 |  | 4 |  | -3 |  |
| **LESS_PRODUCTIVE_EMOTION_IMPAIR** | -3 |  | -26 |  | 87 | * | 8 |  | -3 |  | -2 |  | -4 |  | -6 |  | -5 |  | -3 |  |
| **MARRIEDSTATUS** | -13 |  | 0 |  | -5 |  | -10 |  | 49 | * | -15 |  | 20 |  | -8 |  | -6 |  | -53 | * |
| **NEGATIVE_CONTROL_ANGER** | 1 |  | 76 | * | -1 |  | 2 |  | -3 |  | -5 |  | -4 |  | 0 |  | 11 |  | -1 |  |
| **NEGATIVE_REACTION** | 0 |  | 71 | * | -16 |  | -31 |  | 0 |  | 8 |  | 5 |  | 2 |  | -3 |  | 11 |  |
| **NEGATIVE_UNABLE_CONTROL** | -6 |  | 68 | * | -16 |  | -30 |  | 16 |  | 5 |  | 6 |  | 6 |  | -2 |  | -3 |  |
| **NEGATIVE_UNEXPECTED_EVENTS** | 0 |  | 74 | * | -7 |  | 4 |  | 6 |  | -3 |  | 3 |  | 1 |  | 13 |  | -5 |  |
| **POSITIVE_ATTITUDE_FUTURE** | 9 |  | -19 |  | 2 |  | 68 | * | -6 |  | -13 |  | -3 |  | -9 |  | 10 |  | -1 |  |
| **POSITIVE_CONTROL_IRRITATION** | 2 |  | -5 |  | -1 |  | 64 | * | 10 |  | 14 |  | 10 |  | 19 |  | -23 |  | 0 |  |
| **POSITIVE_COPING_ABILITY** | -1 |  | 67 | * | -11 |  | -16 |  | 11 |  | 1 |  | 3 |  | 10 |  | -22 |  | 3 |  |
| **POSITIVE_HANDLE_PROBLEMS** | 7 |  | -12 |  | 17 |  | 73 | * | -8 |  | -7 |  | -4 |  | 2 |  | -1 |  | 7 |  |
| **POSITIVE_TOPCONTROL** | 6 |  | -35 |  | 17 |  | 62 | * | -2 |  | -11 |  | -8 |  | -17 |  | -4 |  | -6 |  |
| **RT_CONFIDENCE** | -5 |  | 1 |  | -6 |  | -4 |  | 1 |  | 68 | * | 15 |  | 3 |  | 1 |  | -2 |  |
| **SES_PRIMARY_EARNER** | 0 |  | 6 |  | -7 |  | -2 |  | 27 |  | -4 |  | 9 |  | 3 |  | 1 |  | 80 | * |
| **STRESS_EXPOSURE** | 2 |  | 80 | * | -14 |  | -4 |  | 11 |  | -1 |  | -6 |  | 0 |  | -7 |  | 0 |  |
| **STRESS_LEVELHOME** | 6 |  | 11 |  | -14 |  | -11 |  | 54 | * | 20 |  | -14 |  | -5 |  | -5 |  | 9 |  |
| **TROUBLE_CONCENTRATION** | 2 |  | -24 |  | 89 | * | 8 |  | -2 |  | -4 |  | 0 |  | -5 |  | -4 |  | 6 |  |
| **WORK_STATUS** | -6 |  | 0 |  | -7 |  | 3 |  | 16 |  | 12 |  | 70 | * | 2 |  | 16 |  | 5 |  |
| **FEMININITY BEMS** | -1 |  | -7 |  | -3 |  | -3 |  | 1 |  | -11 |  | 25 |  | 67 | * | -7 |  | 25 |  |
| **MASCULINITY BEMS** | -1 |  | 1 |  | 4 |  | 6 |  | -22 |  | -68 | * | 1 |  | 11 |  | -2 |  | -7 |  |

**Supplemental Table 2. Gender-related variables associated with sex in EVA cohort**

|  | **Coefficient Estimate** | **P** |
| --- | --- | --- |
| **Primary responsibility for doing housework** | 1.7459 | <.0001 |
| **Engagement in recreational social activities** | -1.0404 | 0.0006 |
| **Being married or living with partner** | -0.6162 | 0.0278 |
| **Number of hours spent for household chores** | 0.0593 | 0.0002 |
| **Emotional Support Received** | -0.8183 | 0.0005 |
| **Primary Earner Status** | -1.5726 | <.0001 |
| **Level of stress at home** | 0.7166 | 0.0007 |
| **Someone available that you can trust and confide in** | -0.7748 | 0.0036 |

**Appendix B - Rockwood Frailty Index**

**Supplemental Table 3. Items Included into the Frailty Index with Deficits Definition**

| **DOMAINS** | **DEFICIT DEFINITION AND VALUE** | | | | | | | | | | |
| --- | --- | --- | --- | --- | --- | --- | --- | --- | --- | --- | --- |
| **i) COMORBIDITIES** |  | | | | | | | | | | |
| **1. Previous CHD** | YES | | | | | | NO | | | | |
|  | 1 | | | | | | 0 | | | | |
| **2. Heart Failure** | YES | | | | | | NO | | | | |
|  | 1 | | | | | | 0 | | | | |
| **3. Hypertension** | YES | | | | | | NO | | | | |
|  | 1 | | | | | | 0 | | | | |
| **4. Diabetes Mellitus** | YES | | | | | | NO | | | | |
|  | 1 | | | | | | 0 | | | | |
| **5. Dyslipidaemia** | YES | | | | | | NO | | | | |
|  | 1 | | | | | | 0 | | | | |
| **6. Previous Stroke/TIA** | YES | | | | | | NO | | | | |
|  | 1 | | | | | | 0 | | | | |
| **7. Peripheral Vascular Disease** | YES | | | | | | NO | | | | |
|  | 1 | | | | | | 0 | | | | |
| **8. Dementia** | YES | | | | | | NO | | | | |
|  | 1 | | | | | | 0 | | | | |
| **9. COPD** | YES | | | | | | NO | | | | |
|  | 1 | | | | | | 0 | | | | |
| **10. Connective Tissue Diseases** | YES | | | | | | NO | | | | |
|  | 1 | | | | | | 0 | | | | |
| **11. Peptic Ulcer** | YES | | | | | | NO | | | | |
|  | 1 | | | | | | 0 | | | | |
| **12. Chronic Liver Disease** | YES | | | | | | NO | | | | |
|  | 1 | | | | | | 0 | | | | |
| **13. Hemiplegia** | YES | | | | | | NO | | | | |
|  | 1 | | | | | | 0 | | | | |
| **14. Chronic Kidney Disease** | YES | | | | | | NO | | | | |
|  | 1 | | | | | | 0 | | | | |
| **15. BMI** | <18.5>30 | | | 25-30 | | | | | 18.5-25 | | |
|  | 1 | | | 0.5 | | | | | 0 | | |
| **16. Previous Myocardial Infarction** | YES | | | | | | NO | | | | |
|  | 1 | | | | | | 0 | | | | |
| **17. Previous PCI-S** | YES | | | | | | NO | | | | |
|  | 1 | | | | | | 0 | | | | |
| **18. Obstructive CAD** | YES | | | | | | NO | | | | |
|  | 1 | | | | | | 0 | | | | |
| **19. Number of coronary lesions** | 0 | | 1 | | | | | >1 | | | |
|  | 0 | | 0.5 | | | | | 1 | | | |
| **20. Symptomatic presentation** | YES | | | | | | NO | | | | |
|  | 1 | | | | | | 0 | | | | |
| **21. Haemoglobin (g/dL) <13 (Males); <12 (Females)** | YES | | | | | | NO | | | | |
|  | 1 | | | | | | 0 | | | | |
| **22. Ratio NEU/LYM >Median (2.60)** | YES | | | | | | NO | | | | |
|  | 1 | | | | | | 0 | | | | |
| **23. CRP > 0.5 mg/dL** | YES | | | | | | NO | | | | |
|  | 1 | | | | | | 0 | | | | |
| **24. eGFR (CKD-EPI) mL/Min/1.73 m^2^** | >60 | | | | 30-60 | | | | | <30 | |
|  | 0 | | | | 0.5 | | | | | 1 | |
| **25. Albumin (g/dL)** | <2.7 | | 2.7-3.5 | | | | | >3.5 | | | |
|  | 1 | | 0.5 | | | | | 0 | | | |
| **ii) DASI** |  | | | | | | | | | | |
| **26. Take care of yourself, that is, eat, dress, bathe or use the toilet?** | YES | | | | | | NO | | | | |
|  | 0 | | | | | | 1 | | | | |
| **27. Walk indoors, such as around your house?** | YES | | | | | | NO | | | | |
|  | 0 | | | | | | 1 | | | | |
| **28. Walk a block or two on level ground?** | YES | | | | | | NO | | | | |
|  | 0 | | | | | | 1 | | | | |
| **29. Climb a flight of stairs or walk up a hill?** | YES | | | | | | NO | | | | |
|  | 0 | | | | | | 1 | | | | |
| **30. Run a short distance?** | YES | | | | | | NO | | | | |
|  | 0 | | | | | | 1 | | | | |
| **31. Do light work around the house like dusting or washing dishes?** | YES | | | | | | NO | | | | |
|  | 0 | | | | | | 1 | | | | |
| **32. Do moderate work around the house like vacuuming, 3.50 sweeping floors or carrying groceries?** | YES | | | | | | NO | | | | |
|  | 0 | | | | | | 1 | | | | |
| **33. Do heavy work around the house like scrubbing floors or lifting or moving heavy furniture?** | YES | | | | | | NO | | | | |
|  | 0 | | | | | | 1 | | | | |
| **34. Do garden-work like raking leaves, weeding, or pushing a lawn mower?** | YES | | | | | | NO | | | | |
|  | 0 | | | | | | 1 | | | | |
| **35. Have sexual relations?** | YES | | | | | | NO | | | | |
|  | 0 | | | | | | 1 | | | | |
| **36. Participate in moderate recreational activities like golf, bowling, dancing, doubles tennis or throwing a ball?** | YES | | | | | | NO | | | | |
|  | 0 | | | | | | 1 | | | | |
| **37. Participate in strenuous sports like swimming, singles tennis, football, basketball, or skiing?** | YES | | | | | | NO | | | | |
|  | 0 | | | | | | 1 | | | | |
| **iii) Rose Angina Questionnaire** |  | | | | | |  | | | | |
| **38. Have you ever had chest discomfort or pain?** | YES | | | | | | NO | | | | |
|  | 1 | | | | | | 0 | | | | |
| **39. Have you ever felt this pain / discomfort while running or walking uphill?** | YES | | | | | | NO | | | | |
|  | 1 | | | | | | 0 | | | | |
| **40. Have you ever felt this pain / discomfort while walking on the flat?** | YES | | | | | | NO | | | | |
|  | 1 | | | | | | 0 | | | | |
| **iv) PSS-10** |  | | | | | | | | | | |
| **41. In the last month, how often have you been upset because of something that happened unexpectedly?** | Never | Almost Never | | | | Sometimes | | | Fairly Often | | Very Often |
|  | 0 | 0.25 | | | | 0.5 | | | 0.75 | | 1 |
| **42. In the last month, how often have you felt that you were unable to control the important things in your life?** | Never | Almost Never | | | | Sometimes | | | Fairly Often | | Very Often |
|  | 0 | 0.25 | | | | 0.5 | | | 0.75 | | 1 |
| **43. In the last month, how often have you felt nervous and “stressed”?** | Never | Almost Never | | | | Sometimes | | | Fairly Often | | Very Often |
|  | 0 | 0.25 | | | | 0.5 | | | 0.75 | | 1 |
| **44. In the last month, how often have you felt confident about your ability to handle your personal problems?** | Never | Almost Never | | | | Sometimes | | | Fairly Often | | Very Often |
|  | 0 | 0.25 | | | | 0.5 | | | 0.75 | | 1 |
| **45. In the last month, how often have you felt that things were going your way?** | Never | Almost Never | | | | Sometimes | | | Fairly Often | | Very Often |
|  | 0 | 0.25 | | | | 0.5 | | | 0.75 | | 1 |
| **46. In the last month, how often have you found that you could not cope with all the things that you had to do?** | Never | Almost Never | | | | Sometimes | | | Fairly Often | | Very Often |
|  | 0 | 0.25 | | | | 0.5 | | | 0.75 | | 1 |
| **47. In the last month, how often have you been able to control irritations in your life?** | Never | Almost Never | | | | Sometimes | | | Fairly Often | | Very Often |
|  | 0 | 0.25 | | | | 0.5 | | | 0.75 | | 1 |
| **48. In the last month, how often have you felt that you were on top of things?** | Never | Almost Never | | | | Sometimes | | | Fairly Often | | Very Often |
|  | 0 | 0.25 | | | | 0.5 | | | 0.75 | | 1 |
| **49. In the last month, how often have you been angered because of things that were outside of your control?** | Never | Almost Never | | | | Sometimes | | | Fairly Often | | Very Often |
|  | 0 | 0.25 | | | | 0.5 | | | 0.75 | | 1 |
| **50. In the last month, how often have you felt difficulties were piling up so high that you could not overcome them?** | Never | Almost Never | | | | Sometimes | | | Fairly Often | | Very Often |
|  | 0 | 0.25 | | | | 0.5 | | | 0.75 | | 1 |

**Legend:** BMI= Body Mass Index; CAD= Coronary Artery Disease; CHD= Coronary Heart Disease; CKD-EPI= Chronic Kidney Disease Epidemiology Collaboration; COPD= Chronic Obstructive Pulmonary Disease; CRP= C-Reactive Protein; DASI= Duke Activity Status Index; eGFR= estimated Glomerular Filtration Rate; LYM= Lymphocytes; NEU= Neutrophils; PCI-S= Percutaneous Coronary Intervention and Stent; PSS= Perceived Stress Scale; TIA= Transient Ischemic Attack.

**Appendix C - Machine Learning Methods**

The overall process of definition of the ML classification model is reported in **Figure 1** in the paper**.** After the data was pre-processed to obtain the target set, a 75% of the data was used to train the final model and the remaining 25% for evaluating the test performance**. Supplemental Table 5** reports the baselines of train and test cohort. Several classification models were tested (from Logistic Regression to more complex ML models such as Support Vector Machines, and ensemble methods like Random Forest and XgBoost) in order to select the best model for the purpose of the study. For each model, a random search hyperparameter tuning, that has been empirically and theoretically shown to be more efficient than grid search methods, was carried out in order to compute the setting of hyperparameters with the best performance. Given a ML model, a specific hyperparameter setting was sampled from a probability distribution and its performance was assessed by means of a stratified k-fold cross-validation (with k=5). This procedure involves dividing the training set into 5 equal-size folds in a stratified way, that is preserving the distribution of the outcome in each fold (i.e., 67.5% of patients with an obstructive outcome and 32.5% with a non-obstructive outcome). Each ML model is trained over 4 folds and its performance is validated over the remaining fold. This procedure is iterated 5 times to consider as a validation set all the 5 folds available, and the average validation error is computed. The k-fold cross-validation has been shown to reduce the possible bias in the model due to different initial partitioning of the data (as the process is repeated k times using different random partitioning). In the end, the hyperparameters that give the best average validation values of the Key Performance Indicators (KPIs) are chosen and the model is trained on the full training set (75% of data) with the optimal setting. The ML model selected is XgBoost.

**Statistical analysis and Features selection**

As a first step to analyze data, a Pearson correlation matrix was created to exploit the linear correlation between features. The matrix is reported in **Supplementary Figure 1** where the first two rows /columns refer to the output (CAD =obstructive/non-obstructive; ACS = acute / non acute CAD) and the other 18 rows/columns are the features. As we can see from the matrix, the output correlates slightly with all the features, and there is no evidence of some dominance among them. However, there are two features that highly correlate with each other, as expected, which are biological sex and gender score. Since including both those variables with high linear correlation may affect the performance of the model by multicollinearity problem, we left only the gender score in the data set, because it contains more information that could have a role in the disease.

The aim of the research project is to identify the minimal set of features that allows obtaining good performance in the classification of obstructive and non-obstructive CAD and having an explainable behaviour from the bio-clinical point of view. To this aim, we should in principle solve a hard combinatorial problem, performing an extensive enumeration of selected features that due to the large number of combinations will not be used.

Features were selected considering both their importance as displayed by the Shap values tool and the correlation indexes between features, together with the bio-clinical knowledge base on cytokine pathways. The feature selection process is reported in **Supplementary Figure 2**.

At each loop, a model is trained with a given set of features, the KPIs are evaluated both for training and test set and the ranking of the features coming from Shapley tool is considered (Shapley tool displays the features in order of the importance that such feature has in that specific model). If the KPIs do not deteriorate with respect to the values evaluated for the previous model, then we select a reduced number of features among those with the highest SHAP value that seem to explain a bio-chemical process and they are used to train a new XGBoost model. We note that sometimes the ranking of the Shap tool is not strict and different selection options appear to have potentially the same impact. In these cases, even when the KPIs deteriorate we can consider a different features selection without decreasing the overall number. The most promising models are saved. The selection of the final models made using both a KPIs analysis and a bio-clinical interpretation. We report in the **Supplemental Table 6** the full list of setting of features considered in the training of an XGBoost model and their related performances. The final selected model is reported in the first row and it has 9 features that are: gender score, age, frailty index, IL-1β, IL-18, IL-8, IL-23, IL-12p70, IL-33. The performances of this final model on the test set are pretty good and outperform the full features model, thus allowing to discriminate the obstructive CAD to non-obstructive ones with high KPIs. A comparison among the KPIs of the full and selected features model is reported in **Supplemental** **Table 8**. For completeness we also report the values of the KPIs obtained by traditional ML models such as Logistic Regression and Random Forest in **Supplemental Table 9.**

**Key Performance Indicators (KPIs)**

Different metrics were used to assess and benchmark the performance of machine learning models. Since the dataset is imbalanced, the type I errors (False Positive) and type II errors (False Negative) have different meanings, especially from a prognostic point of view. Real outcomes and predictions were summarized in confusion matrices as in **Supplemental Figure 3.**

KPIs used as proxies of the model performance are accuracy, recall and precision obtained on the information provided by the confusion matrix.

The *accuracy* is the fraction of correctly classified instances

$$Accuracy=\frac{TP+TN}{ofinstances}$$

It might represent a misleading indicator because of the imbalanced dataset.

The *recall* is the fraction of patients that are correctly classified as obstructive CAD (true positive).

$$Recall=\frac{TP}{TP+FN}$$

The *precision* represents how often the obstructive patients are correctly classified.

$$Precision=\frac{TP}{TP+FP}$$

This metric is important since high values of precision indicate that the classifier rarely classifies a patient as non-obstructive if it is not.

KPIs are reported for both training and test set, to evaluate if there is overfitting/underfitting or if the model is balanced [Goodfellow, Ian, et al. Deep learning. Vol. 1. No. 2. Cambridge: MIT press, 2016].

The model is subject to underfitting of the training data when it performs poorly on the training data: it happens when the model is not able to capture the relationship between the input data and the outcome. The model is subject to overfitting of the training data when it has high performances in the training data but low ones in the testing data: the model is not able to generalize what learned from the training set to correctly classify new unseen samples.

**Extreme Gradient Boosting**

XGBoost is an optimized application of gradient boosting. It is an ensemble method that puts together a series of weak learners (random trees), where each learner is sequentially trained based on the error made by the previous one [Tianqi Chen and Carlos Guestrin. 2016. XGBoost: A Scalable Tree Boosting System. In Proceedings of the 22nd ACM SIGKDD International Conference on Knowledge Discovery and Data Mining (KDD '16). Association for Computing Machinery, New York, NY, USA, 785–794.]. XGBoost is a specific implementation of the Gradient Boosting method that uses more accurate approximations to find the best tree model. The formation of an XGBoost is an iterative procedure that calculates at each step the best possible subdivision for the k-th tree, listing all the possible structures still available at that point of the path.

XGBoost depends on several hyperparameters that have been set by a random-search tuning. These are:

- max_depth: it determines the maximum number of levels for each tree.
- subsample: it indicates the percentage of samples used per tree. Underfitting could be led with a low value of this parameter.
- colsample_bytree: it indicates the percentage of features used per tree. Overfitting could be led by high values of this parameter.
- n_estimators: maximum number of trees that can be trained before stopping the algorithm.
- learning_rate: value in [0,1] with multiplies each output of XGBoosted trees in order to shrink the contribution of each tree and prevent overfitting.
- gamma: to reduce the sensitivity of the tree to single observations to the prediction. The larger, the more conservative the algorithm is.

Fifty random combinations of the values indicated for these parameters were tested in order to assess which combination of them allowed better performance than the others. We end up with the following setting:

| **Subsample** | **n_estimators** | **min_child_weight** | **'max_depth** | **learning_rate** | **gamma** | **colsample_bytree** |
| --- | --- | --- | --- | --- | --- | --- |
| 0.6 | 800 | 5 | 3 | 0.02 | 0.5 | 0.6 |

**Supplemental Table 4. XgBoost performance on dataset obtaining by elimination of rows with missing values or by imputing missing values (MV) with different standard rules.**

|  | **Training set** | | |  | **Test set** | | | |
| --- | --- | --- | --- | --- | --- | --- | --- | --- |
| **MODEL: XgBoost** | **Accuracy**  **(%)** | **Precision**  **(%)** | **Recall**  **(%)** | **ROC AUC score** | **Accuracy**  **(%)** | **Precision**  **(%)** | **Recall**  **(%)** | **ROC AUC score** |
| **Eliminating patients with MV** | 88.8 | 88.8 | 95.5 | 0.953 | 83.3 | 87.0 | 88.7 | 0.809 |
| **Linear imputation of MV** | 81.5 | 81.8 | 92.9 | 0.899 | 77.6 | 80.6 | 87.7 | 0.801 |
| **Bayesian imputation of MV** | 86.2 | 84.9 | 96.4 | 0.938 | 78.8 | 82.0 | 87.7 | 0.791 |
| **Means imputation of MV** | 79.5 | 80.0 | 92.3 | 0.879 | 76.5 | 78.5 | 89.5 | 0.830 |
| **Median imputation of MV** | 79.5 | 80.3 | 91.7 | 0.879 | 77.6 | 79.7 | 89.5 | 0.827 |
| **Most frequent value imputation of MV** | 80.3 | 80.5 | 92.9 | 0.880 | 77.6 | 78.8 | 91.2 | 0.831 |

**Supplemental Table 5. Baselines of train cohort (n=233 individuals) and test cohort (n=78 individuals).**

| **TRAIN DATA**  **(n=233)** | **Age** | **IL-1β** | **IL-8** | **IL-12p70** | **IL-18** | **IL-23** | **IL-33** | **GENDER SCORE** | **FRAILTY INDEX** |
| --- | --- | --- | --- | --- | --- | --- | --- | --- | --- |
| Median | 68.00 | 0.96 | 15.56 | 2.49 | 105.38 | 0.00 | 46.30 | 0.21 | 0.32 |
| Mean | 66.75 | 2.85 | 31.99 | 5.67 | 188.20 | 10.31 | 106.98 | 0.34 | 0.32 |
| Std | 11.23 | 6.17 | 43.33 | 11.61 | 251.73 | 28.83 | 233.09 | 0.31 | 0.11 |
| Min | 22.00 | 0.00 | 0.00 | 0.00 | 12.69 | 0.00 | 0.00 | 0.01 | 0.10 |
| 25% percentile | 60.00 | 0.00 | 6.68 | 0.00 | 58.97 | 0.00 | 22.60 | 0.06 | 0.24 |
| 50% percentile | 68.00 | 0.96 | 15.56 | 2.49 | 105.38 | 0.00 | 46.30 | 0.21 | 0.32 |
| 75% percentile | 75.00 | 2.67 | 34.38 | 5.41 | 222.87 | 8.80 | 97.90 | 0.60 | 0.40 |
| Max | 89.00 | 52.13 | 227.6 | 112.90 | 2101.6 | 225.33 | 1947.5 | 0.99 | 0.60 |
| **TEST DATA**  **(n=78)** | **Age** | **IL-1β** | **IL-8** | **IL-12p70** | **IL-18** | **IL-23** | **IL-33** | **GENDER SCORE** | **FRAILTY INDEX** |
| Median | 69.00 | 0.99 | 16.71 | 1.79 | 120.01 | 0.00 | 44.3 | 0.23 | 0.28 |
| Mean | 67.79 | 3.80 | 36.84 | 5.22 | 169.54 | 6.79 | 82.16 | 0.34 | 0.30 |
| Std | 10.80 | 7.33 | 47.06 | 8.23 | 162.39 | 15.75 | 100.06 | 0.31 | 0.11 |
| Min | 46.00 | 0.00 | 0.00 | 0.00 | 14.55 | 0.00 | 0.00 | 0.001 | 0.14 |
| 25% percentile | 59.25 | 0.00 | 8.29 | 0.00 | 71.11 | 0.00 | 22.125 | 0.06 | 0.21 |
| 50% percentile | 69.00 | 0.99 | 16.71 | 1.79 | 120.01 | 0.00 | 44.30 | 0.23 | 0.28 |
| 75% percentile | 76.00 | 3.68 | 43.75 | 5.93 | 222.96 | 0.00 | 101.9 | 0.60 | 0.37 |
| Max | 90 | 41.71 | 251.5 | 36.3 | 1005.1 | 78.17 | 528.1 | 0.92 | 0.61 |

**Supplemental Table 6. Full list of setting of 17 features used in the training of an XGBoost model and their related performances. The first row corresponds to the selected 9 features.**

| **1** | **2** | **3** | **4** | **5** | **6** | **7** | **8** | **9** | **10** | **11** | **12** | **13** | **14** | **15** | **16** | **17** |
| --- | --- | --- | --- | --- | --- | --- | --- | --- | --- | --- | --- | --- | --- | --- | --- | --- |
| **Age** | **Gender Score** | **BMI** | **FI** | **IL-1β** | **IFN-α2** | **IFN-γ** | **TNF-α** | **MCP1** | **IL-6** | **IL-8** | **IL-10** | **IL-12p70** | **IL-17A** | **IL-18** | **IL-23** | **IL-33** |

| **Number Features** | **Detailed**  **Features** | **Train** | | | **Test** | | |
| --- | --- | --- | --- | --- | --- | --- | --- |
|  |  | **Accuracy (%)** | **Recall (%)** | **Precision (%)** | **Accuracy**  **(%)** | **Recall**  **(%)** | **Precision (%)** |
| 9 | 1;2;4;5;11;13;15;16;17 | 88.84 | 95.54 | 88.76 | 83.33 | 88.68 | 87.04 |
| 5 | 1;2;4;5;15 | 81.97 | 97.45 | 80.10 | 75.64 | 90.57 | 77.42 |
| 6 | 1;2;4;5;10;15 | 76.39 | 95.54 | 75.76 | 79.49 | 88.68 | 82.46 |
| 7 | 1;2;4;5;13;15;16 | 76.82 | 93.63 | 76.96 | 82.05 | 90.57 | 84.21 |
| 8 | 1;2;4;5;11;13;16;17 | 77.68 | 92.36 | 78.38 | 82.05 | 88.68 | 85.45 |
|  | 1;2;4;5;11;13;15;17 | 74.68 | 91.72 | 75.79 | 79.49 | 84.91 | 84.91 |
|  | 1;2;4;5;11;13;15;16 | 79.83 | 92.99 | 80.22 | 79.49 | 84.91 | 84.91 |
|  | 1;4;5;11;13;15;16;17 | 84.98 | 95.54 | 84.27 | 71.79 | 75.47 | 81.63 |
|  | 1;4;5;10;11;13;16;17 | 74.68 | 94.27 | 74.75 | 74.36 | 83.02 | 80.00 |
|  | 1;2;4;5;11;15;16;17 | 72.53 | 94.27 | 72.91 | 75.64 | 88.68 | 78.33 |
|  | 1;2;4;5;6;15;16;17 | 84.98 | 94.27 | 85.06 | 78.21 | 83.02 | 84.62 |
|  | 1;2;4;5;9;15;16;17 | 81.55 | 94.27 | 81.32 | 83.33 | 88.68 | 86.04 |
|  | 1;2;4;5;9;15;16;17 | 81.55 | 94.27 | 81.32 | 83.33 | 88.68 | 86.04 |
|  | 1;2;4;5;11;13;15;16 | 79.83 | 92.99 | 80.22 | 79.49 | 84.91 | 84.91 |
|  | 1;2;4;5;13;15;16;17 | 87.12 | 95.54 | 86.71 | 80.77 | 88.68 | 83.93 |
| 9 | 1;2;4;10;12;13;15;16;17 | 76.82 | 92.36 | 77.54 | 78.21 | 84.91 | 83.33 |
|  | 1;2;4;5;10;11;13;16;17 | 77.68 | 92.99 | 78.07 | 83.33 | 90.57 | 85.71 |
|  | 1;2;4;5;10;11;13;15;17 | 75.97 | 92.99 | 76.44 | 78.21 | 83.02 | 84.62 |
|  | 1;2;4;5;10;11;13;15;16 | 77.68 | 91.72 | 78.69 | 82.05 | 90.57 | 84.21 |
|  | 1;2;4;5;7;11;13;15;16 | 81.97 | 94.27 | 81.77 | 79.49 | 90.57 | 81.36 |
|  | 1;2;4;5;6;11;13;15;16 | 74.68 | 94.27 | 74.75 | 80.77 | 88.68 | 83.93 |
|  | 1;2;4;5;7;11;15;16;17 | 79.40 | 92.99 | 79.78 | 79.49 | 84.91 | 84.91 |
|  | 1;2;4;5;6;11;15;16;17 | 100.00 | 100.00 | 100.00 | 70.51 | 81.13 | 76.79 |
|  | 1;2;4;5;8;11;15;16;17 | 88.41 | 95.54 | 88.24 | 76.92 | 84.91 | 81.82 |
|  | 1;2;4;5;9;11;15;16;17 | 100.00 | 100.00 | 100.00 | 74.36 | 86.79 | 77.97 |
|  | 1;2;4;5;9;10;15;16;17 | 80.69 | 94.27 | 80.43 | 82.05 | 86.79 | 86.79 |
|  | 1;2;4;5;6;13;15;16;17 | 88.84 | 95.54 | 88.76 | 82.05 | 88.68 | 85.45 |
|  | 1;2;4;5;9;13;15;16;17 | 90.13 | 96.18 | 89.88 | 82.05 | 86.79 | 86.79 |
| 10 | 1;2;4;5;7;8;12;13;15;16 | 84.98 | 94.27 | 85.06 | 80.77 | 84.91 | 86.54 |
|  | 1;2;4;5;11;12;13;15;16;17 | 84.98 | 92.99 | 85.88 | 76.92 | 83.02 | 83.02 |
|  | 1;2;4;5;10;11;13;15;16;17 | 87.98 | 94.90 | 88.17 | 79.49 | 84.91 | 84.91 |
|  | 1;2;4;5;10;11;12;15;16;17 | 72.96 | 90.45 | 74.74 | 79.49 | 84.91 | 84.91 |
|  | 1;2;4;5;7;11;13;15;16;17 | 85.41 | 94.90 | 85.14 | 79.49 | 84.91 | 84.91 |
|  | 1;2;4;5;7;11;12;15;16;17 | 81.12 | 94.27 | 80.87 | 79.49 | 83.02 | 86.27 |
|  | 1;2;4;5;6;11;13;15;16;17 | 100.00 | 100.00 | 100.00 | 71.79 | 83.02 | 77.19 |
|  | 1;2;4;5;8;11;13;15;16;17 | 86.70 | 94.90 | 86.63 | 79.49 | 86.79 | 83.64 |
|  | 1;2;4;5;9;11;13;15;16;17 | 87.98 | 97.45 | 86.44 | 82.05 | 86.79 | 86.79 |
|  | 1;2;4;5;11;12;13;15;16;17 | 84.98 | 92.99 | 85.88 | 76.92 | 83.02 | 83.02 |
| 11 | 1;2;4;5;6;10;12;13;15;16;17 | 89.27 | 96.18 | 88.82 | 82.05 | 88.68 | 85.45 |
|  | 1;2;4;5;9;10;12;13;15;16;17 | 89.27 | 96.82 | 88.37 | 83.33 | 88.68 | 87.04 |
|  | 1;2;4;5;10;11;12;13;15;16;17 | 87.98 | 96.18 | 87.28 | 83.33 | 84.91 | 90.00 |
|  | 1;2;4;5;10;11;12;13;14;15;16 | 74.25 | 96.18 | 73.66 | 80.77 | 90.57 | 82.76 |
|  | 1;2;4;5;10;11;12;13;15;16;17 | 87.98 | 96.18 | 87.28 | 83.33 | 84.91 | 90.00 |
|  | 1;2;4;5;7;10;11;12;15;16;17 | 75.11 | 92.36 | 75.92 | 78.21 | 83.02 | 84.62 |
|  | 1;2;4;5;9;10;11;13;15;16;17 | 76.39 | 94.90 | 76.02 | 80.77 | 86.79 | 85.19 |
| 12 | 1;2;4;5;7;10;11;12;13;15;16;17 | 87.98 | 95.54 | 87.72 | 82.05 | 86.79 | 86.79 |
| 13 | 5;6;7;8;9;10;11;12;13;14;15;16;17 | 87.12 | 95.54 | 86.71 | 70.51 | 83.02 | 75.86 |
| 15 | 1;4;5;6;7;8;9;10;11;12;13;14;15;16;17 | 85.41 | 95.54 | 84.75 | 80.77 | 88.68 | 83.93 |
| 16 | 1;2;4;5;6;7;8;9;10;11;12;13;14;15;16;17 | 76.39 | 95.54 | 75.76 | 79.49 | 86.79 | 83.64 |
| 17 | 1;2;3;4;5;6;7;8;9;10;11;12;13;14;15;16;17 | 75.50 | 96.80 | 74.50 | 78.20 | 88.70 | 81.00 |

**Supplemental Table 7. Comparison of the selected XgBoost model with traditional ML models: Logistic Regression and Random Forest with the selected final features**

|  | **Train set** | | | | **Test set** | | | |
| --- | --- | --- | --- | --- | --- | --- | --- | --- |
| **MODEL** | **Accuracy**  **(%)** | **Precision**  **(%)** | **Recall**  **(%)** | **ROC AUC score** | **Accuracy**  **(%)** | **Precision**  **(%)** | **Recall**  **(%)** | **ROC AUC score** |
| **Logistic Regression** | 76.4 | 78.0 | 90.4 | 0.764 | 69.2 | 74.6 | 83.0 | 0.732 |
| **Random Forest** | 100 | 100 | 100 | 1.00 | 70.5 | 77.8 | 79.2 | 0.741 |
| **XgBoost** | 88.8  : | 88.8 | 95.5 | 0.953 | 83.3 | 87.0 | 88.7 | 0.809 |

**Supplemental Table 8. Comparison among the KPIs of the full features model and selected features model**

|  | **KPIs on the Test Patients** | | |
| --- | --- | --- | --- |
|  | **Accuracy** | **Precision** | **Recall** |
| Model with all the 17 features | 78.20 | 81.00 | 88.70 |
| Selected model with 9 features | 83.33 | 87.04 | 86.68 |

**Supplemental Table 9. Comparison of the selected XgBoost model with traditional ML models: Logistic Regression and Random Forest using the database with the elimination of patients with missing values in the features**

|  |  | **Train set** | | | | **Test set** | | | |
| --- | --- | --- | --- | --- | --- | --- | --- | --- | --- |
| **Features used** | **Model** | **Accuracy**  **(%)** | **Precision**  **(%)** | **Recall**  **(%)** | **ROC AUC score** | **Accuracy**  **(%)** | **Precision**  **(%)** | **Recall**  **(%)** | **ROC AUC score** |
| **The selected final features** | Logistic Regression | 76.4 | 78.0 | 90.4 | 0.764 | 69.2 | 74.6 | 83.0 | 0.732 |
|  | Random Forest | 100 | 100 | 100 | 1.00 | 70.5 | 77.8 | 79.2 | 0.741 |
|  | XgBoost | 88.8 | 88.8 | 95.5 | 0.953 | 83.3 | 87.0 | 88.7 | 0.809 |
| FI gender score  age | Logistic Regression | 71.7 | 74.1 | 89.2 | 0.723 | 74.4 | 77.7 | 88.7 | 0.766 |
|  | Random Forest | 77.3 | 75.0 | 99.4 | 0.800 | 67.9 | 70.6 | 90.6 | 0.846 |
|  | XgBoost | 70.4 | 71.4 | 93.6 | 0.741 | 76.9 | 76.9 | 94.3 | 0.794 |
| BMI  FI  gender score  age | Logistic Regression | 71.2 | 73.9 | 88.5 | 0.724 | 73.1 | 76.7 | 86.8 | 0.756 |
|  | Random Forest | 79.0 | 77.3 | 97.5 | 0.830 | 75.6 | 75.0 | 96.2 | 0.754 |
|  | XgBoost | 76.8 | 74.9 | 98.7 | 0.838 | 73.1 | 72.9 | 96.2 | 0.754 |
| Age  IL-1β  IL-8  IL-12p70  IL-18  IL-23  IL-33 | Logistic Regression | 71.7 | 71.0 | 98.1 | 0.687 | 66.7 | 67.5 | 98.1 | 0.660 |
|  | Random Forest | 76.0 | 74.6 | 97.5 | 0.884 | 71.8 | 72.5 | 94.3 | 0.706 |
|  | XgBoost | 86.3 | 85.7 | 95.5 | 0.922 | 66.7 | 74.5 | 77.4 | 0.718 |
| Age  BMI  IL-1β  IL-8  IL-12p70  IL-18  IL-23  IL-33 | Logistic Regression | 72.5 | 71.6 | 98.1 | 0.681 | 66.7 | 67.5 | 98.1 | 0.684 |
|  | Random Forest | 97.9 | 96.9 | 100.0 | 0.999 | 67.9 | 73.3 | 83.0 | 0.651 |
|  | XgBoost | 87.6 | 86.4 | 96.8 | 0.949 | 74.4 | 78.0 | 86.8 | 0.745 |

**SUPPLEMENTAL FIGURES**

**Supplemental Figure 1. Pearson correlation matrix between the output (obstructive CAD), in the first row/column, and the 18 input features. Values close to -1 indicate a strong negative correlation among the two selected elements, while values close to +1 suggest a strong positive correlation. Values close to 0 indicate no correlation.**


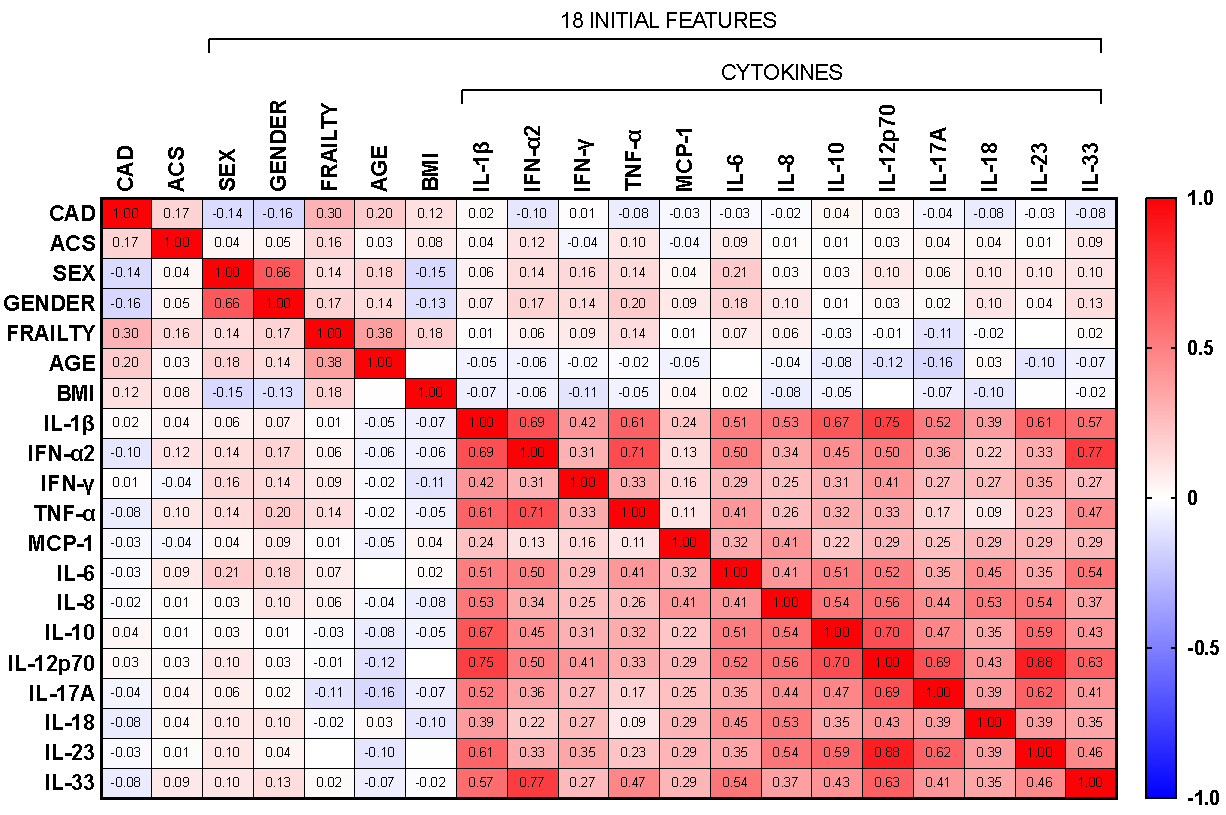


**Supplemental Figure 2. Feature selection process.**


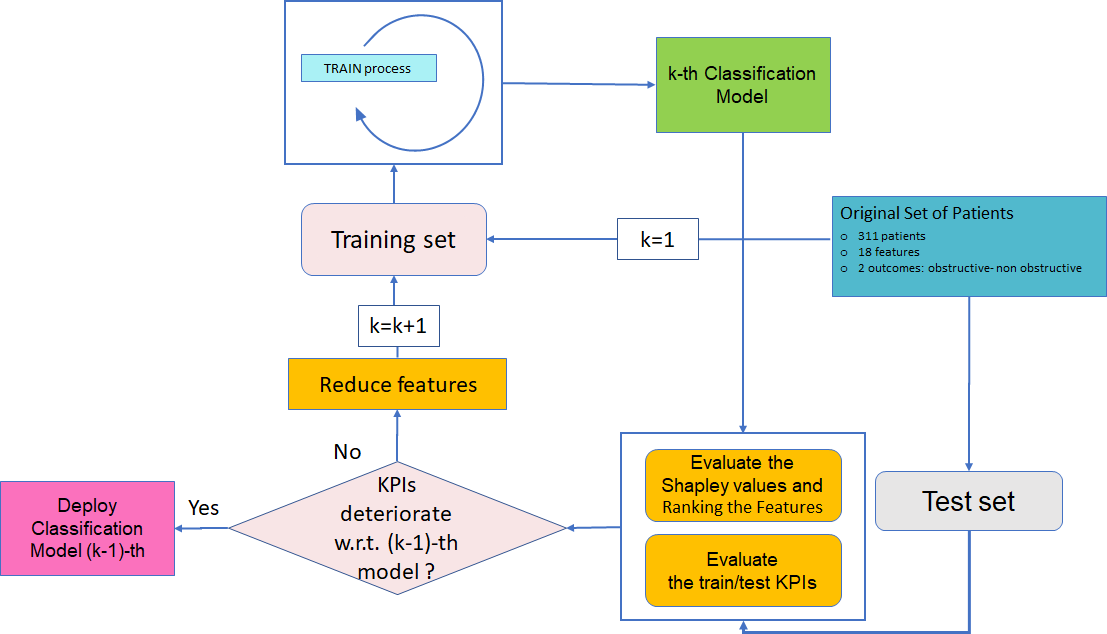


**Supplemental Figure 3. Representative image of a confusion matrix and KPI formulas. TN, FN, FP, TP stand for True Negative, False Negative, False Positive, True Positive, respectively.**


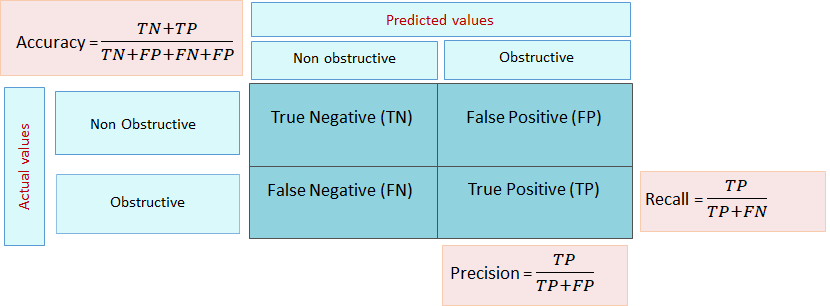


**Supplemental Figure 4. SHAP value plot of the XgBoost model obtained using only the biological data (i.e., concentration of inflammatory cytokines) as input features.**


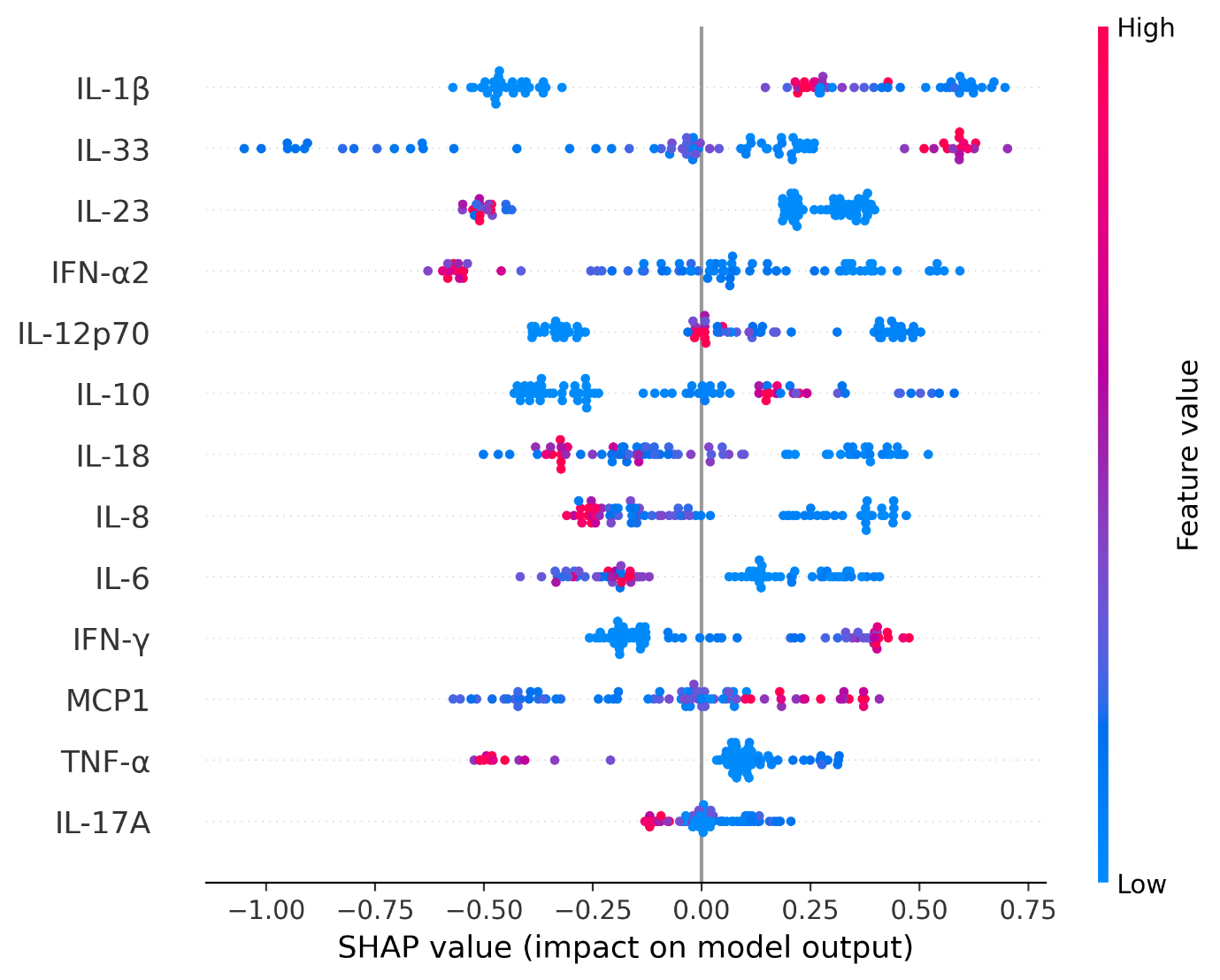


**Supplemental Figure 5. SHAP value plot of the XgBoost model with the following input features: age, gender score, frailty index, 13 cytokines.**


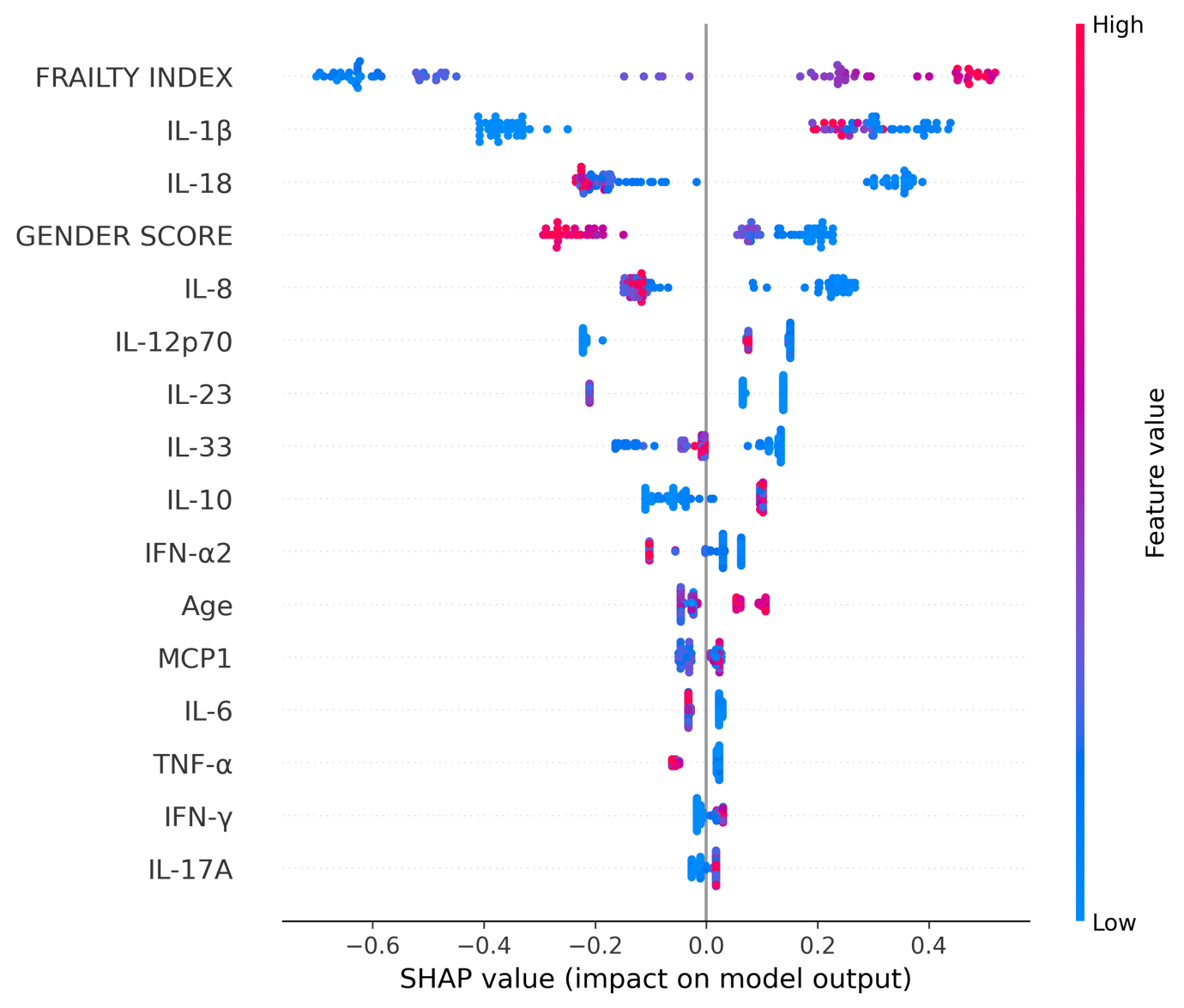


**Supplemental Figure 6. SHAP value plot of the best-performing XgBoost model with the addition of IL-6 among the input features.**


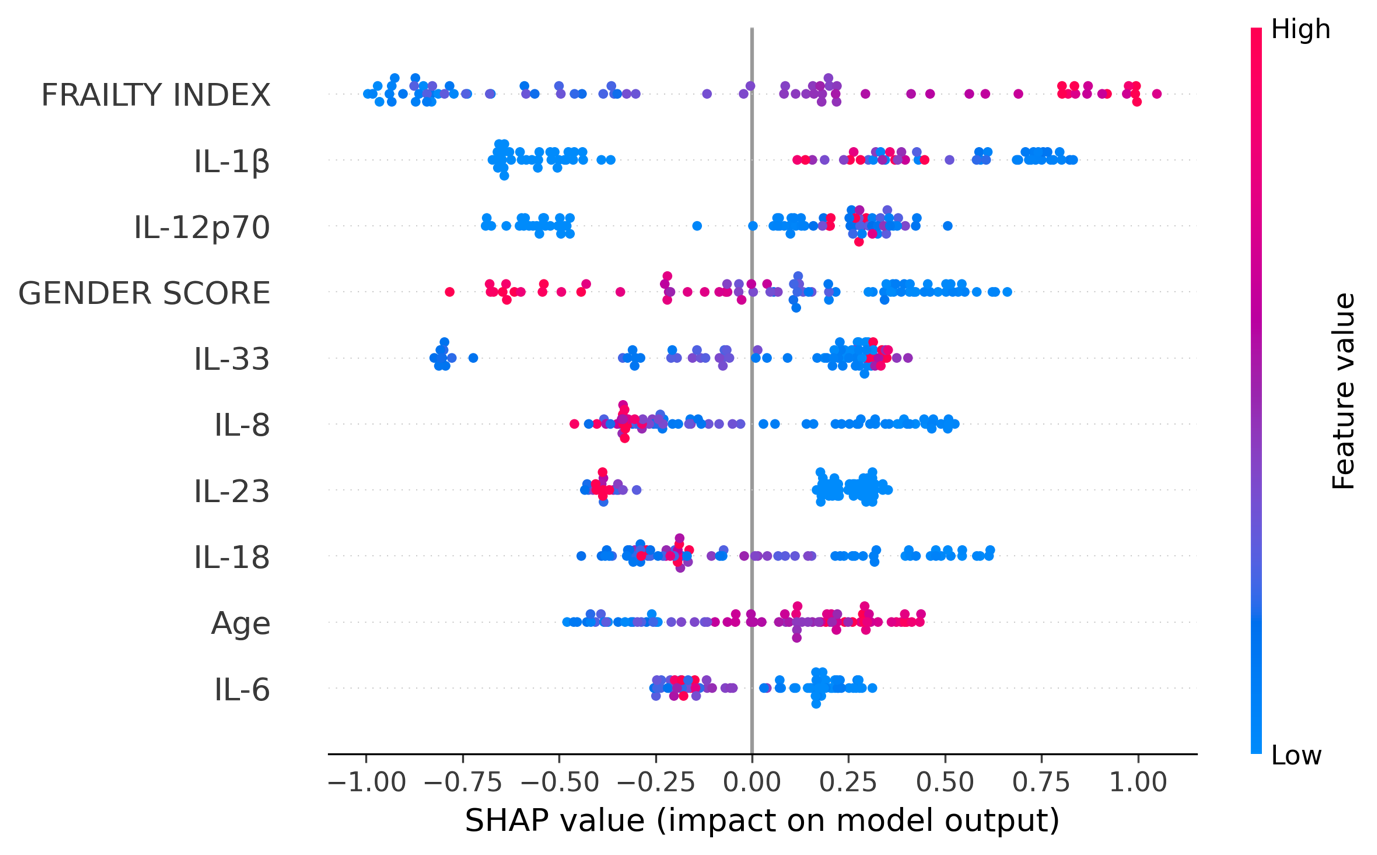


**Supplemental Figure 7. Comparisons of the ROC curve between Logistic Regression (LR), XgBoost (XgB) and Random Forest (RF) using the selected final features (gender score, age, frailty index, IL-1β, IL-18, IL-8, IL-23, IL-12p70, IL-33)**


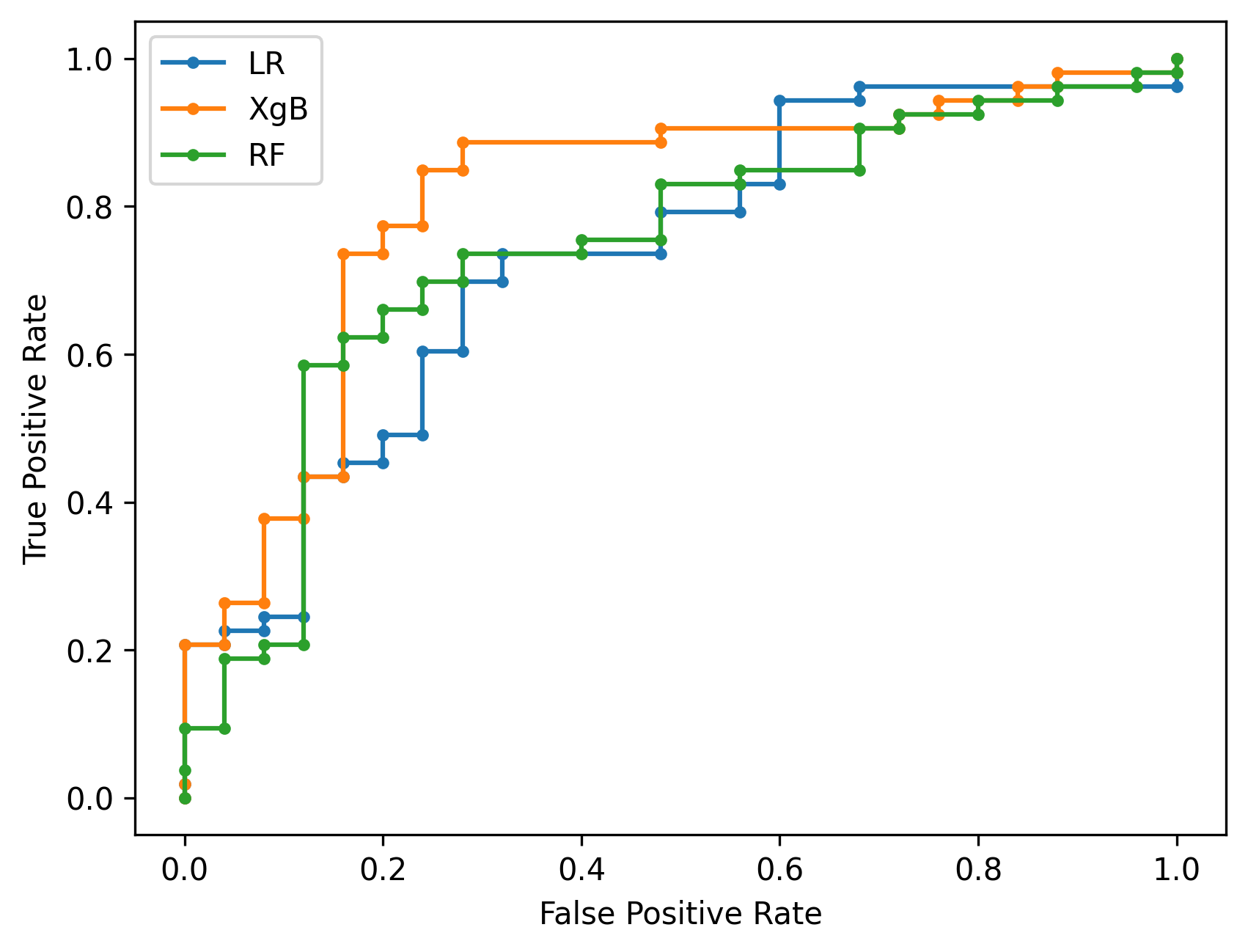


**Supplemental Figure 8. SHAP Plots representing the machine learning prediction of Obstructive and Non-Obstructive CAD only among the acute or stable patients within our cohort.**


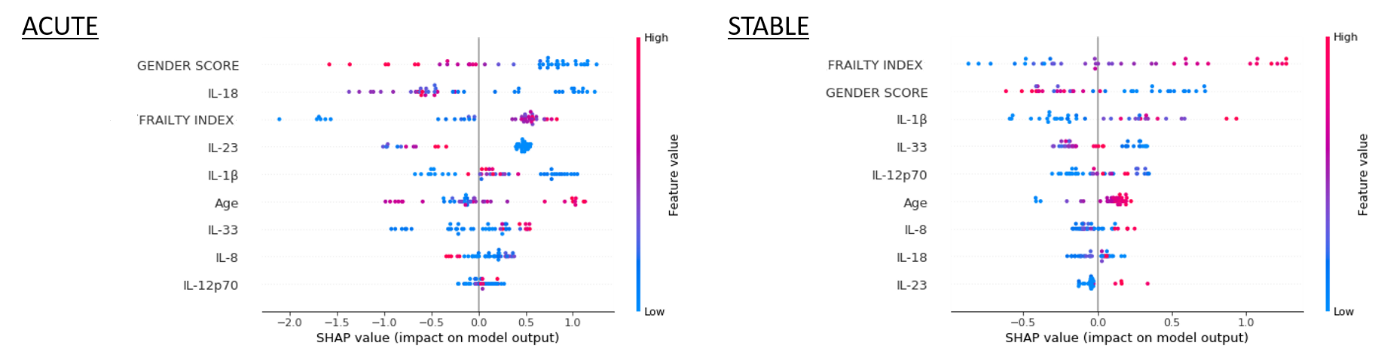


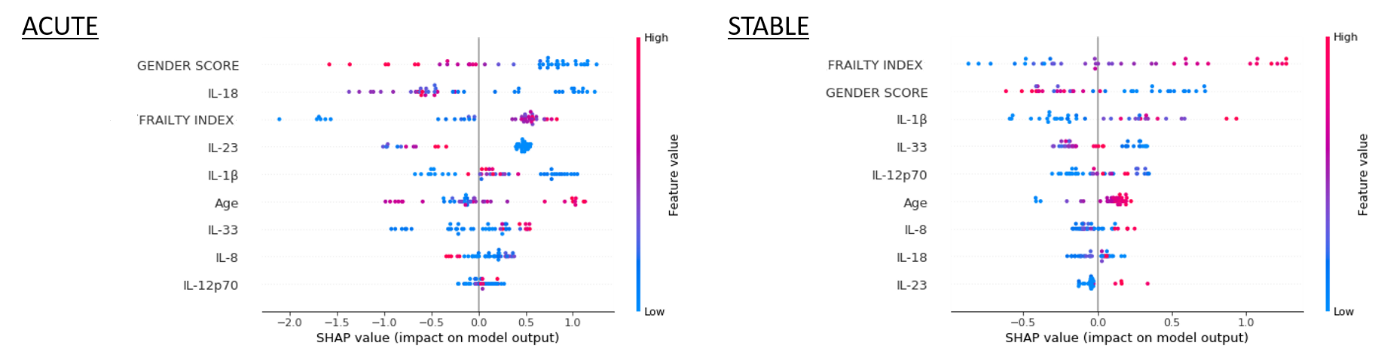

Supplement: Supplementary file 1 — Supplementary file1 (DOCX 943 KB) [file 392_2023_2193_MOESM1_ESM.docx]
